# *In vitro* elucidation of the crucial but complex oxidative tailoring steps in rufomycin biosynthesis enables one pot conversion of rufomycin B to rufomycin C†

**DOI:** 10.1039/d1cc04794a

**Published:** 2021-10-15

**Authors:** Gustavo Perez Ortiz, John D. Sidda, Emmanuel L. C. de los Santos, Catherine B. Hubert, Sarah M. Barry

**Affiliations:** Department of Chemistry, Faculty of Natural, Mathematical & Engineering Sciences, Britannia House, 7 Trinity St London SE1 1DB UK sarah.barry@kcl.ac.uk; School of Life Sciences, University of Warwick, Gibbet Hill Road Coventry CV4 7AL UK

## Abstract

The antimycobacterial peptides, rufomycins, have their antibiotic activity conferred by oxidative tailoring of the cyclic peptide. Here we elucidate the roles of cytochrome P450s RufS and RufM in regioselective epoxidation and alkyl oxidation respectively and demonstrate how RufM and RufS create a complex product profile dependent on redox partner availability. Finally, we report the *in vitro* one pot conversion of rufomycin B to rufomycin C.

## Abstract 2

Cytochrome P450s RufS and RufM catalyse regioselective and stereoselective epoxidation and alkyl oxidation respectively, in a complex series of reactions in the biosynthesis of the antimycobacterial peptides, rufomycins.

## Graphical abstract

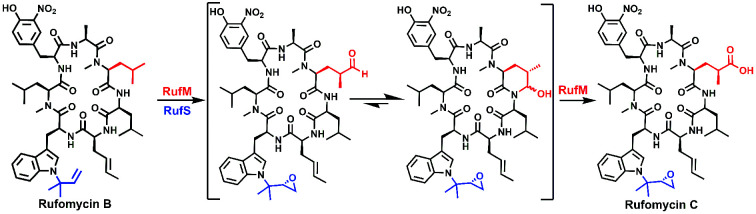

Cytochromes P450 (CYPs) are a large family of heme dependent redox enzymes that activate molecular oxygen to catalyze an array of transformations including oxidative C–C bond formation, hydroxylation and epoxidation.^1^ CYPs have diverse roles in many organisms including biosynthetic and degradative pathways. CYP catalysed oxidation is a frequent feature of non-ribosomal peptide, polyketide and terpene biosynthesis, including modifications to biosynthetic precursors, growing polyketide or non-ribosomal peptide chains or post-cyclisation scaffolds. Such modifications are crucial in the biosynthesis of several clinically used antibiotics including vancomycin, rifampicin and erythromycin.^2^ While the most common reaction catalysed by CYPs is hydroxylation, biosynthetic CYPs, despite a highly conserved tertiary structure, demonstrate exceptional chemical versatility including sequential oxidation, nitration, C–C bond formation, *N*-glycosylation and decarboxylation.^2^ The discovery of new oxidative chemistry, combined with their ability to activate inert C–H bonds on both small and highly complex molecules with exceptional regio and stereoselectivity, has also led to renewed interest in CYPs as potential biocatalysts.^3–5^

In the biosynthesis of the non-ribosomal peptides, rufomycins (also known as ilamycins), CYPs introduce epoxide and carbonyl moieties vital for biological activity (Fig. 1 and ESI,† Fig. S5).^6^ Rufomycins have anticancer activity but have attracted recent interest due to their selective activity against *Mycobacterium tuberculosis (Mtb)*, the causative agent of TB and a leading cause of death from a single infectious agent.^7–9^ Multidrug resistance to frontline antibiotics, rifampicin and isoniazid, is rising, leading to a need for new TB antibiotics.^9,10^ Rufomycins, like the structurally similar cyclomarins, are covalent inhibitors of the *Mtb* protease ClpC1. Binding is facilitated *via* ring opening of the epoxide moiety.^11,12^ Rufomycins are produced as a mixture of variously oxidised derivatives by several *Streptomyces* strains^8,13,14^ (ESI,† Fig. S5). NRPS, RufT, assembles the core cyclic peptide rufomycin B **1** (ESI,† Fig. S6).^6,15^ The two crucial tailoring steps, epoxidation and alkyl oxidation, are catalysed by CYPs, RufS and RufM (Fig. 1). Genetic experiments by Ma *et al.* in *Streptomyces SCSIO ZH16* showed that knocking out *ilaL* (*rufM* homologue, 99.7% identity) produced epoxidised rufomycin **2** while an *ilaR* (*rufS* homologue, 99.6% identity) mutant, produced rufomycins **8–10**, which are not epoxidised, indicating that IlaL and IlaR activity is not dependent on the action of the other enzyme, but not indicating a preferred order (Fig. 1).^15^ RufM/IlaL is thus proposed to catalyse sequential two electron oxidations to generate the aldehyde and carboxylic acid, but genetic experiments cannot rule out the possibility of other enzyme(s) catalysing some oxidative steps. If RufM/IlaL is responsible for all oxidations, the aldehyde intermediate **3** must be released from the RufM/IlaL active site, where it spontaneously cyclises to give the cyclic hemiaminal **4**/**5** (Fig. 1). It has not been shown if **4**/**5** are intermediates or dead ends in the RufM catalytic cycle. These questions and the roles of RufM and RufS in introducing key functionality, motivated us to investigate them *in vitro.*

**Fig. 1:**
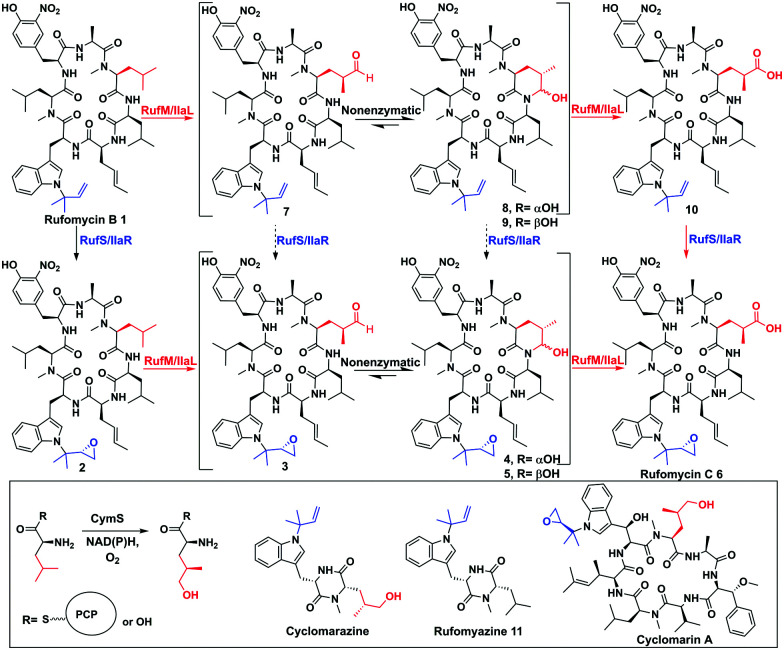
Biosynthetic steps to rufomycins and cyclomarins. Top: Oxidative tailoring of rufomycin B by RufS and RufM or homologues IlaR and IlaL. Steps previously proven (black solid arrows),^15^ this work (red solid), not demonstrated by experiment (black dashed). Inset: Proposed role of CymS in cyclomarin biosynthesis, structures of shunt metabolites rufomyazine, cyclomarazine and antibiotic cyclomarin A.

Rufomycins B **1**, A **4**/**5** and C **6** and rufomyazine (Fig. 1) were isolated from *S. atratus* DSM41673 as both substrates and standards for RufM and RufS *in vitro* reactions (ESI,† Fig. S7–S10).^16^ Identification of rufomycins from *S. atratus* culture extracts was facilitated by feeding ^13^C-2-l-leucine, resulting in triple-^13^C-labelled rufomycins and singly labelled rufomyazine **11** (ESI,† Fig. S11 and S12). Rufomycins **1**, **4**, **5**, **6**, identified by LC-HRMS and characteristic absorbance at 222, 282 and 355 nm, were observed in the same relative abundance as previously reported (ESI,† Fig. S4).^15^ Rufomycin C **6** and rufomyazine **11** were isolated in sufficient quantities for NMR characterisation (ESI,† Fig. S13 and S14).^15,16^ Interestingly, under these growth conditions, rufomyazine is more abundant than any rufomycins (ESI,† Fig. S4). Rufomyazine is analogous to cyclomarazine, a shunt metabolite in cyclomarin biosynthesis, and may result from offloading by a type II thioesterase from module 2 of RufT,^17^ following stalling of the dipeptide on module 2 due to the absence of l-3-nitrotyrosine on module 3 (ESI,† Fig. S6).^16,17^

With substrates and standards in hand, we investigated the function of both CYPs. RufS (and homologue IlaR) has high identity to CymV (51% identity) from cyclomarin biosynthesis, which has been shown to catalyse epoxidation of the prenylated tryptophan moiety (Fig. 1).^17^ RufS was overproduced in *E. coli* as an N-His_6_ tagged fusion protein with a Soret band at 420 nm (Fig. 2A). To demonstrate its activity, RufS was incubated with rufomycin B **1**, NADPH and redox partners ferredoxin (Fd) and ferredoxin reductase (Fr) (Spinach, Aldrich). Analysis of the reactions by LC-HRMS showed conversion of rufomycin B **1** to a single new product, not detected in *S. atratus* culture, but corresponding to epoxidised rufomycin **2** (Fig. 2B and ESI,† Table S8).

**Fig. 2:**
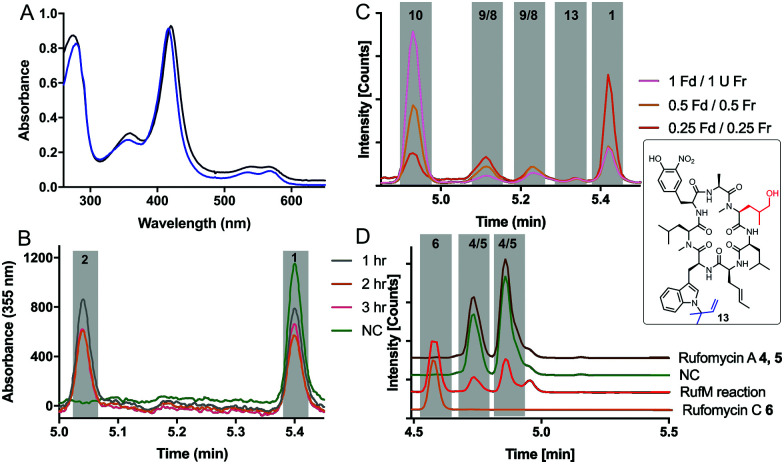
RufS and RufM characterisation: (A) UV-visible spectra of RufS (black) and RufM (blue). (B) LC analysis of RufS with rufomycin B **1** over time (C) LC-HRMS analysis of RufM reactions with **1** with decreasing concentrations of redox partners Fd, Fr. (D) LC-HRMS analysis of reactions of Rufomycin A **4**/**5** with RufM. **4**/**5** and **6** standards were isolated from *S. atratus* cultures.

Subsequently, RufM was produced as an N-terminal His_6_ tagged SUMOylated fusion protein, with a Soret band at 417 nm (Fig. 2A). RufM is 44% identical to the putative δ-leucinyl hydroxylase CymS in cyclomarin A biosynthesis.^17^ However, based on the presence of oxidised leucine in the shunt metabolite, cyclomarazine, CymS is proposed to catalyse hydroxylation of leucine or PCP bound leucine rather than the cyclomarin cyclic peptide (Fig. 1).^17^ To assess its activity, RufM was incubated with rufomycin B **1**. LC analysis shows 3 new peaks (Fig. 2C). HRMS indicates that the major product is the carboxylic acid **10** (ESI,† Table S9), and the two smaller peaks correspond to aldehyde/hemiaminal intermediates **7**, **8**, **9** (Fig. 2C). Pleasingly, the relative proportions of the rufomycins detected in RufM assays, correspond to those observed by Ma *et al.* from culture extracts of the *ilaL* (*rufM* homologue) mutant.^15^ Interestingly, a primary alcohol is not observed under these conditions, nor has an alcohol intermediate been isolated from cultures of *S. atratus* strains DSM41673 or SCSIO-ZH16. However, Zhou *et al.*, reported an alcohol containing rufomycin isolated from *Streptomyces* strain MJM3502, indicating that RufM may produce, and release, an alcohol intermediate.^14^ We hypothesised that the availability of electron donor proteins, Fr and Fd, may influence the product profile. When the concentration of both electron donors was reduced (0.5, 0.25) the proportion of intermediates **8**/**9** increased (Fig. 2C). Additionally, a small peak with mass corresponding to the alcohol **13** is observed (ESI,† Table S10). We then asked if RufM, on releasing the aldehyde intermediate, can rebind it and convert it to rufomycin C **6**. Incubation of RufM with hemi-aminal and epoxide containing rufomycin A **4**/**5**, produced rufomycin C **6**, demonstrating that RufM can accept rufomycin A **4**/**5** and rufomycin B **1** as substrates (Fig. 2D and ESI† Table S11). Interestingly, conversion of rufomycin A **4**/**5** to rufomycin C **6** is incomplete. Aldehyde **3** is in equilibrium with hemiaminal **4**/**5**.^15^ RufM is likely to preferentially bind the aldehyde or corresponding gem-diol, and slow interconversion between these species may result in slow binding to RufM and low conversion. This is supported by our reaction of **4**/**5** with semicarbazide, to trap intermediate aldehyde **3**, which gave just 40% conversion after 72 hours (ESI,† Fig. S15).^18^

Having demonstrated the activity of both RufM and RufS in isolation, we aimed to prove that the products of these reactions were intermediates in the biosynthesis of Rufomycin C **6** and to investigate if there was a preferred order of reaction. This had the added benefit of proving the identity of intermediate products as they could be converted to rufomycin C **6** for which we have a standard. Thus, oxidation of rufomycin B **1** to rufomycin C **6** was carried out using sequential reactions where rufomycin B **1** was oxidised by one enzyme, the products extracted and analysed, then used as substrate(s) for reaction with the second enzyme (Fig. 3).

**Fig. 3:**
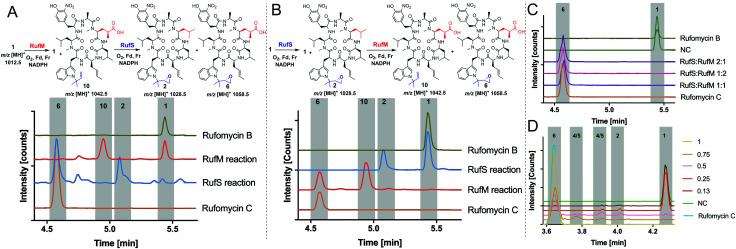
Sequential and one pot oxidation reactions of RufS and RufM with rufomycin B **1**. (A) Oxidation by RufM followed by epoxidation by RufS. Analysis by LC-HRMS. Traces are EICs of 1012.58 *m*/*z*, 1028.58 *m*/*z*, 1026.56 *m*/*z*, 1042.56 *m*/*z* and 1058.55 *m*/*z*. (B) Oxidation of rufomycin B **1** by RufS followed by RufM, analysis by LC-HRMS shown as in A. All reactions contain rufomycin B **1** (40 μM), 1 h, 30 °C in Tris (25 mM, pH 8), ferredoxin (Fd) and ferredoxin reductase (Fr) and NADPH (1 mM). Rufomycin B (green) and rufomycin C (orange) are standards purified from *S. atratus* culture. (C) LC-HRMS analysis of 1 pot reactions of RufS and RufM with rufomycin B **1** (D) LC-HRMS analysis of one pot reactions of RufS and RufM with rufomycin B **1** with various ratios of Fd/Fr relative to RufS/RufM concentration.

Analysis of the RufM reaction shows the presence of remaining rufomycin B **1** and the fully oxidized carboxylic acid product **10** with low levels of intermediates (Fig. 3A). Following incubation of the extracted mixture with RufS, rufomycin B **1** is almost completely converted to epoxide **2** and carboxylic acid **10** is converted to rufomycin C **6** (Fig. 3A and ESI,† Table S12). Similarly, RufS was incubated with rufomycin B **1**, and the products extracted, then used as substrates for RufM. Analysis shows total conversion of both epoxidised rufomycins by RufM after 60 minutes to produce carboxylic acid **10** and rufomycin C **6** (Fig. 3B). Epoxide **2** is thus confirmed for the first time as a substrate for RufM (ESI,† Table S13).

The results indicate that RufS and RufM accept several rufomycin derivatives and indicate a preferred order of RufS followed by RufM. We tested RufM/RufS promiscuity further, incubating them separately with rufomyazine **11**, which contains both moieties modified in rufomycin, however the differences in size and conformation of rufomyazine were unsurprisingly, not tolerated by either enzyme, and no oxidation products were detected (ESI,† Fig. S16).

Finally, we investigated if the *in vivo* product profile is replicated when both enzymes are present. Incubation of RufM and RufS (1 : 1 ratio) with rufomycin B **1** showed complete conversion to rufomycin C **6** (Fig. 3C and ESI,† Table S14). The result is consistent for different enzyme ratios. No intermediates were detected, indicating a greater affinity of both enzymes for the intermediates over rufomycin B **1**. Lowering concentrations of electron donors (Fd/Fr) led to very modest increases in intermediates **2**, **4**/**5** (Fig. 3D and ESI,† Table S15).

Taken together, our results confirm that, like CymV in cyclomarin biosynthesis, RufS performs the vital epoxidation of the prenylated l-tryptophan moiety.^15,17^ We unambiguously show that RufM sequentially oxidises a leucine residue to form hemiaminal and carboxylic acid products. Furthermore, our data demonstrates that alcohol and aldehyde intermediates are released and the aldehyde at least, can rebind. Our data in combination with the accepted mechanism of CYP hydroxylation, involving H abstraction and oxygen rebound, leads us to propose a complex catalytic cycle for RufM (Fig. 4A).^19^ Other CYPs catalyse sequential oxidations including TxtC, which hydroxylates an aliphatic carbon, releases the product and rebinds it in a different orientation to oxidise an aromatic carbon.^19,20^ Substrate reorientation is not required for RufM, as the same carbon is oxidised. SaAcmM (42% identity to RufM) catalyzes the sequential oxidation of an l-proline moiety in actinomycin biosynthesis. Both the 4-hydroxyproline (actinomycin X0) and 4-oxoproline (actinomycin X2) derivatives were detected, indicating like RufM, product release prior to a second round of oxidation.^21^ However, what determines whether a CYP catalyses single or multiple oxidations at a single position is not known.^19^ Sequence alignment shows that it is not due to differences in the catalytically important I helix, as while RufM does not contain the conserved motif, SaAcmM does (Fig. 4B).^22^

**Fig. 4:**
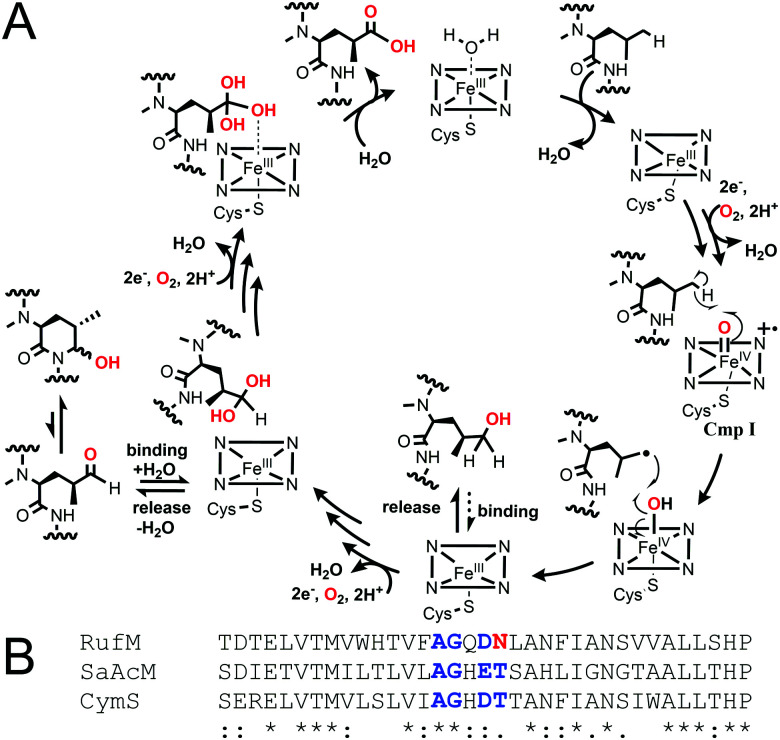
(A) Proposed catalytic cycle of RufM based on this work and accepted CYP oxygen rebound hydroxylation mechanism.^1^ Cmp I = compound I, active oxidising intermediate. (B) Aligned I helix sequences (Clustal Omega) of RufM, CymS and SaAcM. I helix boundary defined by SaAcM structure (5NWS^21^). Conserved motif = AGxD/ET.

We have however demonstrated that release of partially oxidised intermediates is enhanced by reduced availability of electron donors. Differences in rufomycin oxidation product profile between *in vivo* and *in vitro* RufM reactions could result from a combination of factors including secretion, *in vivo*, of reactive, potentially toxic, aldehydes/hemiaminals **3–5** from the cell as a possible resistance mechanism. Differences in *rufM*/*rufS* expression levels may play a role, although changes in RufM/RufS ratios tested here, did not result in a change in product profile. Thus based on our data, the most significant factor in explaining differences between *in vivo* and *in vitro* oxidation is likely to be the use of non-native electron transport donors, as also suggested for differences observed in CYP (PtlI) catalysed sequential oxidation in pentalenolactone biosynthesis.^23^ This result illustrates the possibility of modulating the nature of CYP products by manipulating electron transfer and warrants further study.^24^

While the one-pot reaction of RufM and RufS with Rufomycin B **1**, produced a different product profile to bacterial culture, the unexpected formation of a single product is welcome (Fig. 3C). It represents clean conversion, under mild conditions, of a complex cyclic peptide *via* four, two electron oxidations and the concomitant introduction of two new stereocentres.

CYPs are increasingly attractive to industry as biocatalytic agents thanks to advances such as directed evolution, designed enzymes, ancestral sequence reconstruction and use of inexpensive cofactor surrogates.^25–28^ However, our work underscores the difficulties of predicting substrate preference or chemistry catalysed by CYPs, even where they have high sequence identity to enzymes of known function. Greater understanding is needed to facilitate development of more CYPs as catalysts for functionalization of high value compounds.

We thank the MRC (MC_PC14105 v.2) & BBSRC (BB/P019811/1) for grants to SMB, the Mexican National Council of Science & Technology (CONACyT)/Mexican Secretary of Energy (SENER) & KCL for scholarships to GPO & CBH respectively. ES was supported by the BBSRC & EPSRC (BB/M017982/1). We thank the KCL Centre for Biomolecular Spectroscopy (Wellcome Trust, 202767/Z/16/Z) for high field NMR.

## Conflicts of interest

There are no conflicts to declare.

## Supplementary Material

CC-057-D1CC04794A-s001
