# Supplementary material for: In vitro elucidation of the crucial but complex oxidative tailoring steps in rufomycin biosynthesis enables one pot conversion of rufomycin B to rufomycin C
Source: Chem Commun (Camb). 2021 Oct 15;57(89):11795–8. doi: 10.1039/d1cc04794a (PMC8577248; doi:10.1039/d1cc04794a)
Supplement: CC-057-D1CC04794A-s001 [file CC-057-D1CC04794A-s001.pdf]

Electronic Supplementary Material (ESI) for Chemical Communications.
This journal is © The Royal Society of Chemistry 2021

Supplementary Information.

In vitro Elucidation of the Crucial but Complex Oxidative Tailoring Steps in Rufomycin Biosynthesis enable one pot conversion of Rufomycin B to Rufomycin C.

Gustavo Perez Ortiz[a], John D. Sidda[a], Emmanuel L. C. De Los Santos[b], Catherine B. Hubert[a] and Sarah M. Barry[a*]

[a] *Department of Chemistry, Faculty of Natural, Mathematical and Engineering Sciences, King's College London, Britannia House, 7 Trinity Street, London, SE1 1DB, UK*

[b] *School of Life Sciences, University of Warwick, Gibbet Hill Road, Coventry, CV4 7AL, UK*

* To whom correspondence should be addressed. Email: sarah.barry@kcl.ac.uk

Table of Contents

Supplementary methods..5

Growth and Maintenance of Streptomyces atratus DSM41673 5

Isolation and Purification of rufomycin and rufomyazine 5

LC-HRMS method 6

Calibration curve for rufomycin concentration calculation 6

Feeding experiments with ^{13}C-2-L-Leucine 7

Condensation reaction of aldehyde rufomycin intermediate with semicarbazide 7

Cloning RufS to create overexpression plasmid GPO151-S001 (His_6-RufS plasmid)......................... 7

Amplification of rufM from S. atratus genomic DNA and cloning to create GPO151-M002 overexpression plasmid (His_6-SUMO-RufM plasmid) 8

Overexpression and purification of RufM and RufS 9

Measuring the activity of RufM and RufS 10

Supplementary Figures 11

Supplementary Tables...26

Supporting references 33

List of Figures

Supplementary Fig. S1. Plasmid map of GPO151-S001. 11

Supplementary Fig. S2. Plasmid map of GPO151-M002. 12

Supplementary Fig. S3. SDS PAGE gels of recombinant RufS and RufM. 12

Supplementary Fig. S4. Relative abundance of rufomycin compounds in S. atratus crude extract.. 13

Supplementary Fig. S5. Structures of previously reported natural occurring rufomycins Produced by S. atratus ATCC14046. [5] 14

Supplementary Fig. S6. Biosynthesis of rufomycins and rufomyazine. 15

Supplementary Fig. S7. HRMS analysis of rufomycin B **1**. 16

Supplementary Fig. S8. HRMS analysis of rufomycin A **4** & **5**................................. 17

Supplementary Fig. S9. HRMS analysis of rufomycin C **6**....................................... 18

Supplementary Fig. S10. High resolution extracted Ion Chromatogram of rufomyazine from *S. atratus* cultures in R5 media incubated for 6 days. 19

Supplementary Fig. S11. Rufomycin C 6 feeding experiment with ^{13}C-2-L-leucine.. 20

Supplementary Fig. S 12 Rufomyazine Feeding experiment with ^{13}C-2-L-leucine. .. 20

Supplementary Fig. S13. ^{1}H NMR spectrum of rufomycin C **6** in CD_3OD at 700 MHz. 22

Supplementary Fig. S14. ^{1}H NMR spectrum of rufomyazine **11** in CD_3OD at 400 MHz. 23

Supplementary Fig. S 15. Condensation Reaction of Rufomycin B **1** with Semicarbazide.. 24

Supplementary Fig. S16. Reaction of RufM and RufS with rufomyazine. 25

Table of Tables

Supplementary Table S1. ^{1}H NMR spectroscopic data of rufomycin C **6** and the reported data for the same compound... 21

Supplementary Table S2. The Prep-HPLC mobile phase gradient profile used for purification of the crude rufomycin extract, 20mL min^{-1} flow rate............................. 26

Supplementary Table S3. The analytical HPLC mobile phase gradient profile used for the purification of the fraction of rufomycin extract, 1mL min^{-1} flow rate and LCMS. Formic acid was used instead of TFA in LCMS runs. 26

Supplementary Table S4. HPLC mobile phase gradient profile used for HRMS, 0.400 mL min-1 flow rate. 27

Supplementary Table S5. Primers used in the study.. 27

Supplementary Table S6. PCR cycle parameters for the amplification of the PCR fragments of *rufM* from genomic DNA. 28

Supplementary Table S7. PCR cycle parameters for the amplification of the PCR fragments used during the cloning *rufM* to produce plasmid GPO151-M002. 28

Supplementary Table S8. Epoxidation of rufomycin B **1** by His$_6$-RufS. 28

Supplementary Table S9. Oxidation of rufomycin B **1** by His$_6$-SUMO-RufM. 29

Supplementary Table S10. Oxidation of rufomycin B **1** by His$_6$-SUMO-RufM. 29

Supplementary Table S11. Oxidation of rufomycin A **4/5** by His$_6$-SUMO-RufM. 30

Supplementary Table S12. Sequential reaction 1: oxidation reaction of rufomycin **1** by RufM followed by epoxidation reaction by RufS. 30

Supplementary Table S13. Sequential reaction 1: epoxidation reaction of rufomycin **1** by RufS followed by oxidation reaction by RufS. 31

Supplementary Table S14. RufM and RufS simultaneous reactions with rufomycin **1**. 31

Supplementary Table S15. RufM and RufS simultaneous reactions with rufomycin **1** and different concentrations of electron donours Fd and Fr. 32

Supplementary methods.

Growth and Maintenance of *Streptomyces atratus* DSM41673

Streptomyces atratus DSM41673 was purchased as lyophilized mycelia from LGC standards. Plates of ISP4[1] were inoculated with 20 µL of bacterial spore stocks and incubated at 30° C for 10 days. Sterile water was added to each plate (9 mL to each 25 mL plate), the spores resuspended with a spreader and transferred to a 50 mL falcon tube. Spores were separated by vortex for 1 minute and filtered through cotton wool to remove any large bits of agar. The suspension was centrifuged at 3000 rpm for 5 minutes. The supernatant was removed, leaving approximately 1000 µL of water. The spores were resuspended again and an equal volume of 50% glycerol sterile was added to a final concentration of 25% of glycerol. The spore stocks were aliquoted and flash freeze with liquid nitrogen and were stored at -80 °C.

Isolation and Purification of rufomycins and rufomyazine

For partial purification of rufomycin an Agilent 1260 Preparative HPLC was used. The crude extract was injected onto an Agilent Zorbax C18 reverse phase column (100 x 21.2 mm, 5 µm particle size) at a flow rate of 20 mL min^{-1} (Supplementary Table S2). The UV absorbance of the eluent was monitored at 222 nm, 282 nm and 355 nm. 10 ml fractions were collected. The fractions containing the rufomycin compounds were concentrated by lyophilisation.

The semi-purified pellet was then resuspended in 50:50 HPLC grade acetonitrile and water, centrifuged (14,000 rpm for 10 minutes) and injected onto an analytical reverse phase column Agilent Zorbax C8 column (150 x 4.6 mm, 5 µm particle size) with a flow rate of 0.5 mL min^{-1} to purify the compounds further (Supplementary Table S3). The analytical HPLC used was an Agilent Technologies 1260 Infinity II LC. The UV absorbance of the eluent was monitored at 222 nm, 282 nm and 355 nm. The compound was collected manually, avoiding the contaminants. The solvent was removed again by lyophilisation and the pure compound was stored at -80°C. Pure

samples were used for NMR, LCMS and HRMS analysis and bioassays. The LC-MS used was an Advion Expression Compact mass spectrometer with Agilent 1260 Infinity HPLC with a reverse phase Agilent Zorbax C8 column using the same condition used for the analytical HPLC purification but with formic acid instead of TFA in both Buffer A and B. (Supplementary Table S3)

LC-HRMS method

Samples of purified compounds were resuspended in 50:50 HPLC grade acetonitrile and water, centrifuged (14,000 rpm for 10 minutes) and characterized by LC-HRMS using a Waters Acquity UPLC-Class I equipped with an ACQUITY UPLC BEH C8, 1.7 µm, 2.1 x 50 mm column and connected to a Mass Spectrometer Waters Xevo-G2-XS QT. Column temperature was 60 ºC. Buffer A was water + 0.1% formic acid, Buffer B was acetonitrile + 0.1% formic acid, flow rate was 0.400 mL min^{-1}. (Supplementary Table S4). The UV absorbance of the eluent was monitored at 222 nm, 282 nm and 355 nm.

Calibration curve for rufomycin concentration calculation

To estimate the relative abundance of rufomycins, a calibration curve was created using fmoc-3-nitro-L-tyrosine monitoring the absorbance at λmax 355 nm. It was assumed that the extinction coefficients of fmoc-3-nitro-L-tyrosine and the 3 rufomycin compounds at 355 nm are the same. Solid amino acid was weighed and dissolved in 70% DMSO to obtain a 10 mM stock solution. The stock solution was then used to make lower concentration solutions. 50% acetonitrile in water was used to complete the volume, preparing calibration solutions of 10, 25, 50, 100 and 250 μM. this process was then repeated 2 more times to create the 3 repeats. The repeats were run using the same method as the *S. atratus* crude extract samples using an Agilent Zorbax C18 reverse phase column (100 x 21.2 mm, 5 µm particle size) at a flow rate of 20 mL min^{-1} (Supplementary Table S2, Fig. S4).

Feeding experiments with ^{13}C-2-L-Leucine

S. atratus spores (20 µL) were used to inoculate 25 mL plates of SMMS enriched with 10 mM ^{13}C-2-L-leucine. Cultures were grown for 7d at 30 °C and metabolites extracted as detailed previously. For LC-MS analysis, each sample was diluted by 10 in 50:50 HPLC grade water/acetonitrile and analysed (Supplementary Table S3, S4, Fig. S11).

Condensation reaction of rufomycin A 4/5 with semicarbazide

To confirm the formation of an intermediate aldehyde during the enzyme catalyzed oxidation of rufomycin, a condensation reaction was carried out to capture the aldehyde with semicarbazide [2]. Rufomycin (40 µM final conc.) was incubated at room temperature with semicarbazide (2.5 mM) in Tris buffer (25 mM, pH 8) for 72 hrs. Final volume 100 mL. The reaction was extracted with ethyl acetate and the pellet reconstituted in 80 µL of water. The samples were analysed via LC-HRMS. (Supplementary Table S4, Fig. S15)

Cloning *rufS* to create overexpression plasmid GPO151-S001 (His_6-RufS plasmid)

A pET151/D-TOPO plasmid containing the gene *rufS* was purchased from Thermo Fischer (GeneArt). (Supplementary Fig. S1). This plasmid (GPO151-S001) was used to transform One Shot Top 10 chemically competent cells (Invitrogen) according to manufacturer's instructions. Colonies were isolated in Luria-Bertani (LB) medium plates[3] containing 50 µg mL^{-1} carbenicillin. Positive clones were confirmed by restriction digest using the enzymes BglII, BamHI and SspI (Supplementary Fig. S1) and by sequencing (GATC Biotech).

Amplification of *rufM* from *S. atratus* genomic DNA and cloning to create GPO151-M002 overexpression plasmid (His_6-SUMO-RufM plasmid)

S. atratus DSM41673 genomic DNA was isolated according to manufacturer's instructions using an Omega E.N.Z.A Soil DNA kit from 1cm^2 of *S. atratus* culture plate grown on ISP4 for 7 days.

Specific primer pair were designed to specifically amplify *rufM* by PCR. PCR mix contained sterile H_2O (37.5 µL), DMSO (2.5 µL), dNTPs (10 µM each, 2 µL), Roche High Fidelity DNA polymerase (3.5 U, 1 µL), buffer 2 (5 µL), gDNA (1 µL), and *rufM* primers (1 µL each of 100 µM stocks) (Supplementary Table S5). PCRs were performed using an Eppendorf Mastercycler Nexus gradient eco thermocycler using conditions in table (Supplementary Table S6).

The reactions were analysed by gel electrophoresis and PCR products were purified by QIAquick Gel Extraction Kit (Qiagen) and DNA concentration was determined using a Labtech Nanodrop ND-8000 spectrophotometer and diluted accordingly to fall within the desired range of 1-5 ng µL^{-1} for cloning into pET151 as directed by the Invitrogen TOPO cloning manual. 1 µL of the diluted PCR product was mixed with sterile water and the other components of the kit and incubated for 5 min as directed in the TOPO cloning manual (Thermo Fischer).

2-3 µL of the cloning mixture was added to TOP10 cells (25 µL), storing on ice for 30 min before being incubated (42 °C, 40 s) immediately followed by addition of 250 µL ice-cold LB. The cells were incubated (37 °C, 180 rpm, 60 min) and plated out onto LB agar plates with 100 µg mL^{-1} final concentration of carbenicillin. Selection plates were used to isolate single colonies, which were picked and used to innoculate LB (10 mL, 100 µg mL^{-1} carbenicillin) and incubated at 37 °C for 16 h. Plasmids purified from these overnight cultures and correct clones were confirmed by PCR with primers used for cloning the gene from gDNA (Supplementary Table S5), restriction digests and Sanger sequencing (GATC biotech).

rufM was subsequently subcloned by PCR and assemble into a pET15b plasmid (a gift from Dr Manuel Muller [4]), (SUMOpET15b) using the NEBuilder High-Fidelity DNA Assembly Cloning Kit from New England Biolabs®. The primers were designed using the NEBuilder Assembly Tool (http://nebuilder.neb.com) (Supplementary Table S5).

Each PCR reaction was prepared in triplicate with the following reagents: 5x Q5 reaction buffer (10 µL), dNTP mix (10mM each, 1 µL), primers (2.5 µL each of 10 µM stocks), 5x Q5 GC enhancer (10 µL) Q5 High-Fidelity DNA polymerase (New England Biolabs) (0.5 µL) and DNA template (1 µL, 3 ng) and water up to 50 µL, respectively following the method in Supplementary Table S7. On completion the PCR reactions were loaded on agarose gels and the PCR products were separated by electrophoresis (90V for 1hr). The bands of the correct

size were cut, and the DNA fragment was extracted using the QIAquick Gel Extraction Kit (Qiagen). The DNA concentration was determined using a Labtech Nanodrop ND-8000 spectrophotometer.

The assembly reaction was carried out using a 1:2 ratio plasmid:gene insert. The reaction was set in duplicates and was prepared with the following reagents: 1 µL plasmid (35 ng), 1 µL gene insert (70 ng), 5 µL HiFi DNA Assembly Master Mix (New England Biolabs) and 2.5 µL water. The reaction was incubated 45 minutes at 50 °C. 2 µL of the assembly reaction was used to transform NEB 5-alpha competent *E. coli* cells following manufacturer's instructions. Colonies were isolated in LB plates containing 50µg mL^{-1} carbenicillin. Positive clones were confirmed by restriction digest using the enzymes EcoRI and SspI and by sequencing (Genewiz) (Supplementary Fig. S2). The resulting plasmid was named GPO151-M002 and encodes the fusion protein His_6-SUMO-RufM (Supplementary Fig S2).

Overexpression and purification of RufM and RufS

GPO151-S001 or GPO151-M002 was used to transform *E. coli* BL21-DE3 (Invitrogen) and/or Tuner-DE3 (Sigma-Aldrich), respectively according to manufacturer's instructions. Single colonies were grown in 10 mL of LB liquid media (50µg mL^{-1} carbenicillin) and incubated overnight at 30°C with shaking (220 rpm). 3 mL of the overnight culture were added to 500mL of pre-warmed LB (50µg mL^{-1} carbenicillin) and incubated further at 37° C with shaking (230 rpm).

Cultures (OD_{600} 0.8-1) were cooled to 15°C and induced with isopropyl-β-D-thiogalactopyranoside (IPTG) (0.5 mM final conc.) and supplemented with iron (II) sulfate heptahydrate (final con. 1 mM) and 5-aminolevulinic acid (final conc. 5 mM). His_6-RufS cultures were incubated for 42 hrs at 15°C with shaking (230 rpm). His_6-SUMO-RufM cultures were incubated for 16h at 15°C with shaking (230 rpm).

Cells were harvested by centrifugation (4200 rpm, 20 min, 4°C). The pellet was resuspended in the minimum volume of Buffer A (20 mM Tris buffer, 100 mM NaCl, 20 mM imidazole, 10% glycerol) with the addition of dithiothreitol (DTT) (final conc. 0.5mM), Pepstatin A (final conc. 1 µg/mL per), DNAse A (final conc. 0.2mg/ mL) and Complete™ protease inhibitor cocktail (1 tablet per 2 L of culture) (Sigma). Cell lysis was carried out on a cell disruptor IXT4A (Constant System LTD) with a pressure of 25 kPa, 4° C. The lysate was centrifuged (18,000 rpm) for 42 min at 4°C. The lysate

was applied to a 5 mL His-Trap™ Fast Flow nickel affinity column (GE) equilibrated with Buffer A and connected to ÄKTA Pure Chromatography System (GE). His tagged proteins were eluted with Buffer B (20 mM Tris buffer, 100 mM NaCl, 200 mM imidazole, 10% glycerol). Fractions containing the protein of interest were concentrated to a volume less than 5 mL and injected onto a HiLoadTM 16/600 Superdex 200pg size exclusion chromatography column (GE) equilibrated with Buffer C (20 mM Tris buffer, 100 mM NaCl, 10% glycerol), Proteins were eluted with buffer C (His_6-RufS (47.9 kDa), after 81 mL and His_6-SUMO-RufM (56.1 kDa), after 76 mL. The fractions containing the protein were concentrated, mixed with an equal volume of Buffer D (20 mM Tris buffer, 100 mM NaCl, 30% glycerol) and divided into 50 µL aliquots, flash frozen and stored at -80°C (Supplementary Fig S3).

Measuring the activity of RufM and RufS

His_6-RufS or/and His_6-SUMO-RufM (20 µM In Tris buffer 25 mM, pH 8) was added ferredoxin (final conc. 45 µM) and ferredoxin reductase (final conc. 0.08 U) from *Spinacia oleracea* (Sigma), rufomycins in DMSO (40 µM) to a final volume of 100 µL. NADPH (1 mM) was added to initiate the reaction.

Reactions were incubated from 30 minutes to 3 hours at 30 ºC with shaking (800 rpm) in a ThermoMixer C (Eppendorf). Negative controls were carried out as above but RufS/RufM were heat inactivated (>100 ºC, 15 minutes) prior to addition. After incubation, reactions were extracted with ethyl acetate (2 x 500 µL), evaporated to dryness and resuspended in 100 µL 50:50 water:acetonitrile and analysed by LC-HRMS. The samples were analysed by LC-HRMS as above.

For consecutive reactions, after extracting the first reaction and drying the solvent, the pellet was resuspended in 3 µL of DMSO and this was used in place of substrate in the next reaction. After the second reaction, the samples were extracted and analyzed by LC-HRMS as above.(Table S4)

Supplementary Figures

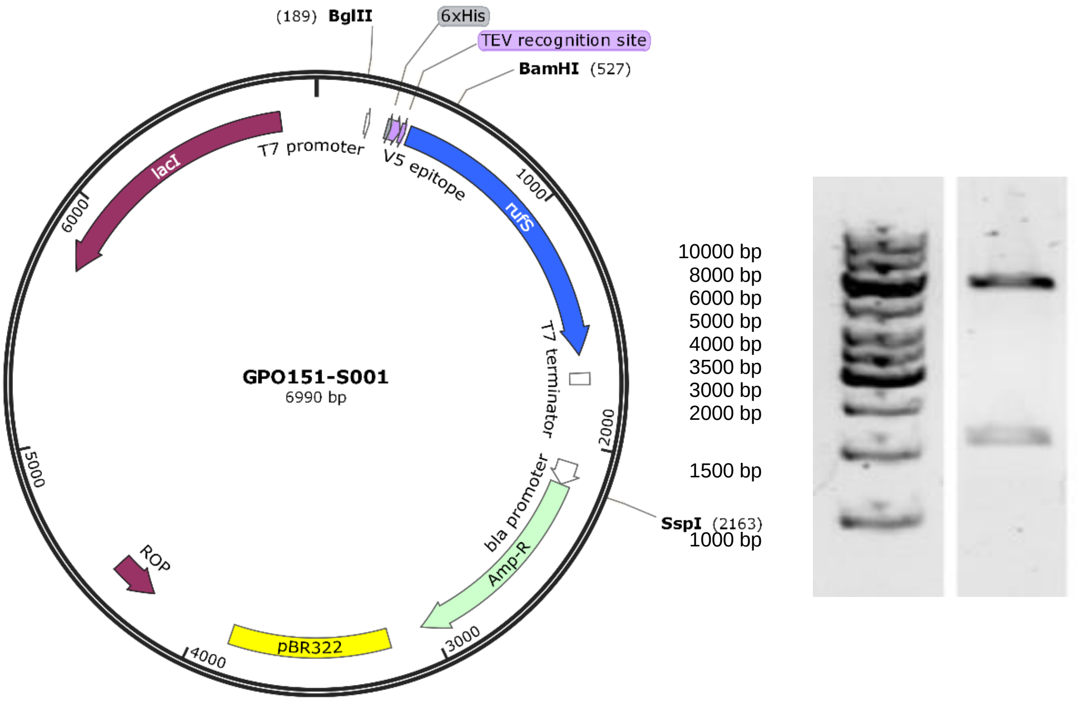

Supplementary Fig. S1. Plasmid map of GPO151-S001.

The map shows the relative location of the gene *rufS* and structural features, for example, an ampicillin resistance gene (AmpR), used for selection. The plasmid incorporates a 6x polyhistidine tag for protein purification with HisTrap columns, a TEV cleavage site and T7 promoter and lac repressor protein (*lacI*) for IPTG-induced gene expression. Restriction digest of GPO151-S001 showing the two bands of expected sizes 5016 and 1974 bp in the presence of BglII and SspI.

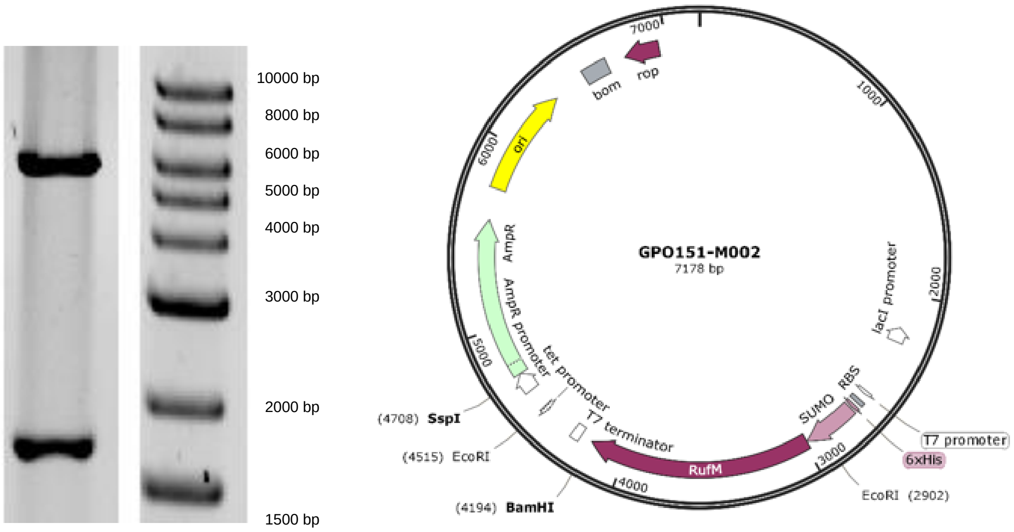

Supplementary Fig. S2. Plasmid map of GPO151-M002.

Restriction digest of GPO151-M002 showing the two bands expected of 5565 and 1613 bp, respectively, in the presence of restriction enzyme EcoRI. Plasmid map of GPO151-M002 showing the relative location of the gene rufM and the Small Ubiquitin-like Modifier (SUMO) solubility partner tag, ampicillin resistance gene AmpR, a 6x polyhistidine tag for protein purification with HisTrap columns, a TEV cleavage site and T7 promoter and lac repressor protein (LacI).

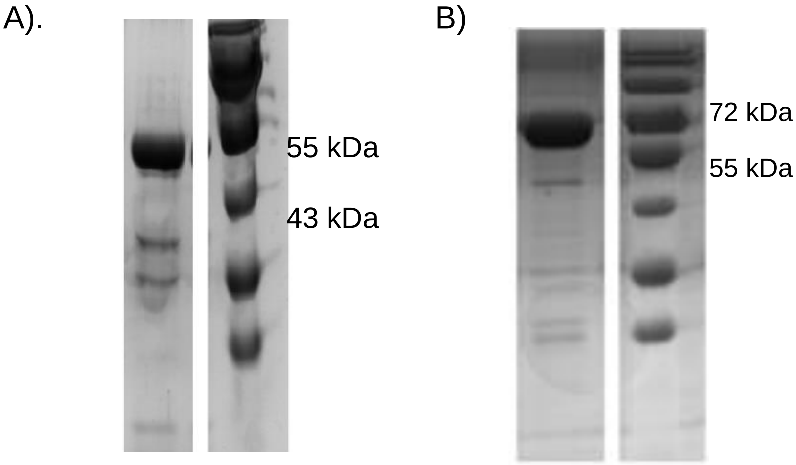

Supplementary Fig. S3. SDS PAGE gels of recombinant RufS and RufM.

A) SDS PAGE of purified recombinant His_6-RufS (47.9 kDa). B) SDS PAGE of purified recombinant His_6-SUMO-RufM (56.1 kDa).

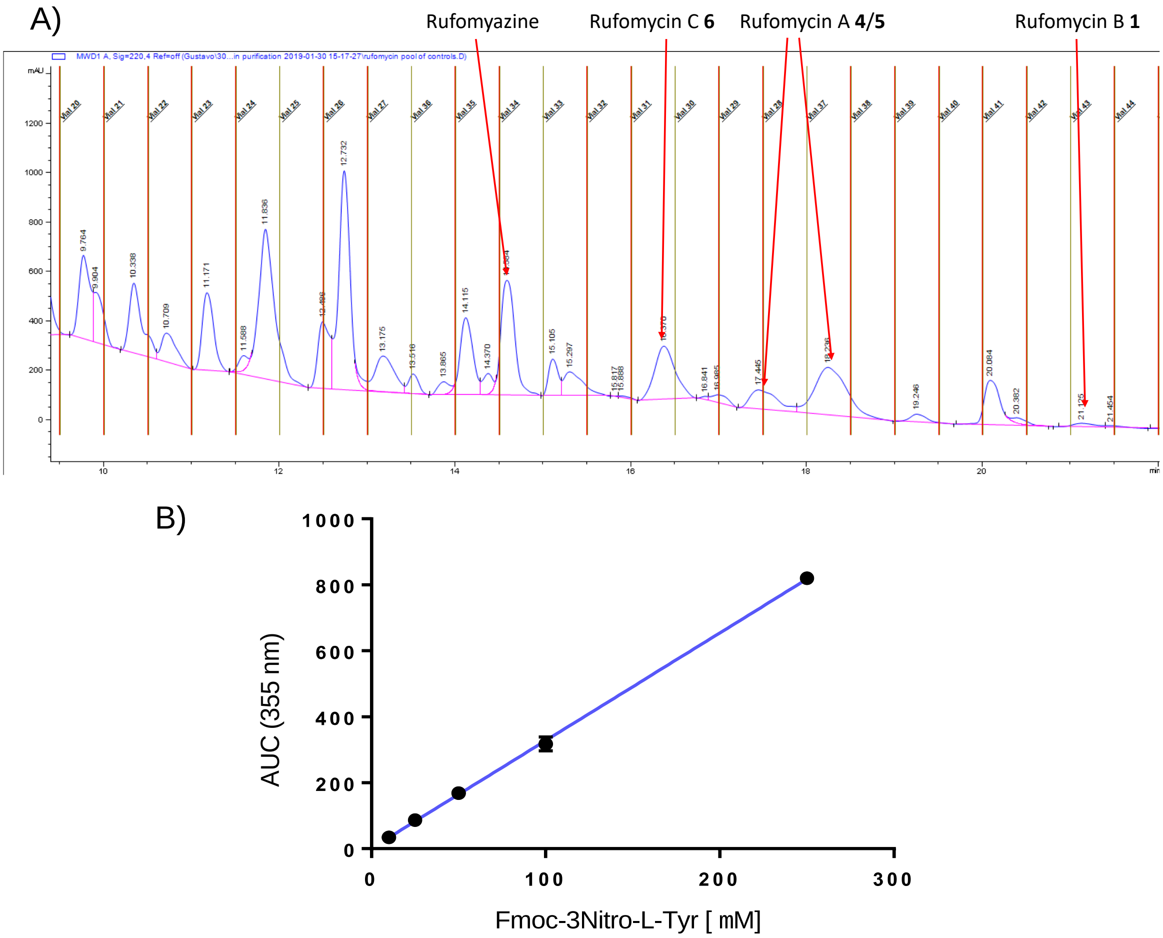

Supplementary Fig. S4. Relative abundance of rufomycin compounds in *S. atratus* crude extract.

A) HPLC spectrum (222 nm) of rufomycin crude extracted from *S. atratus* R5 plates incubated 6 days at 30º C. B) To estimate the relative abundance of rufomycins, a calibration curve was created using fmoc-3-nitro-L-tyrosine monitoring the absorbance at λ_{max} 355 nm. It was assumed that the extinction coefficients of fmoc-3-nitro-L-tyrosine and the 3 rufomycin compounds at 355 nm are the same. The calculated yields of rufomycins in *S. atratus* culture crude extract are: for rufomycin B 0.13 mg per litre of culture; for rufomycin A is 1.82 mg per litre of culture; and for rufomycin C is 1.67 mg per litre. The relative abundance of rufomycin B **1** is 3.7%, rufomycin A **4/5** is 50.2% (12% first peak and 38.2% second peak) and 46.1% for rufomycin C **6**.

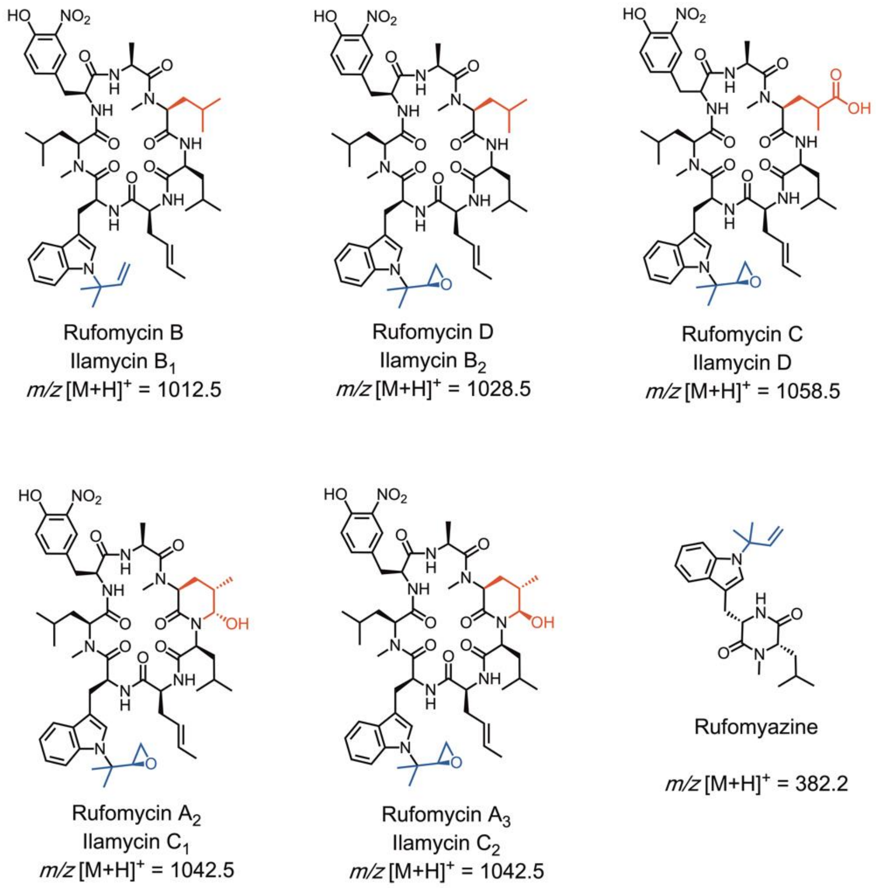

Supplementary Fig. S5. Structures of previously reported natural occurring rufomycins Produced by *S. atratus* ATCC14046. [5]

CYP RufM introduces the sequential oxidation of one *N*-methylated L-leucine residue (red) whereas CYP RufS the epoxidation of the *N*-prenylated L-tryptophan residue (blue).

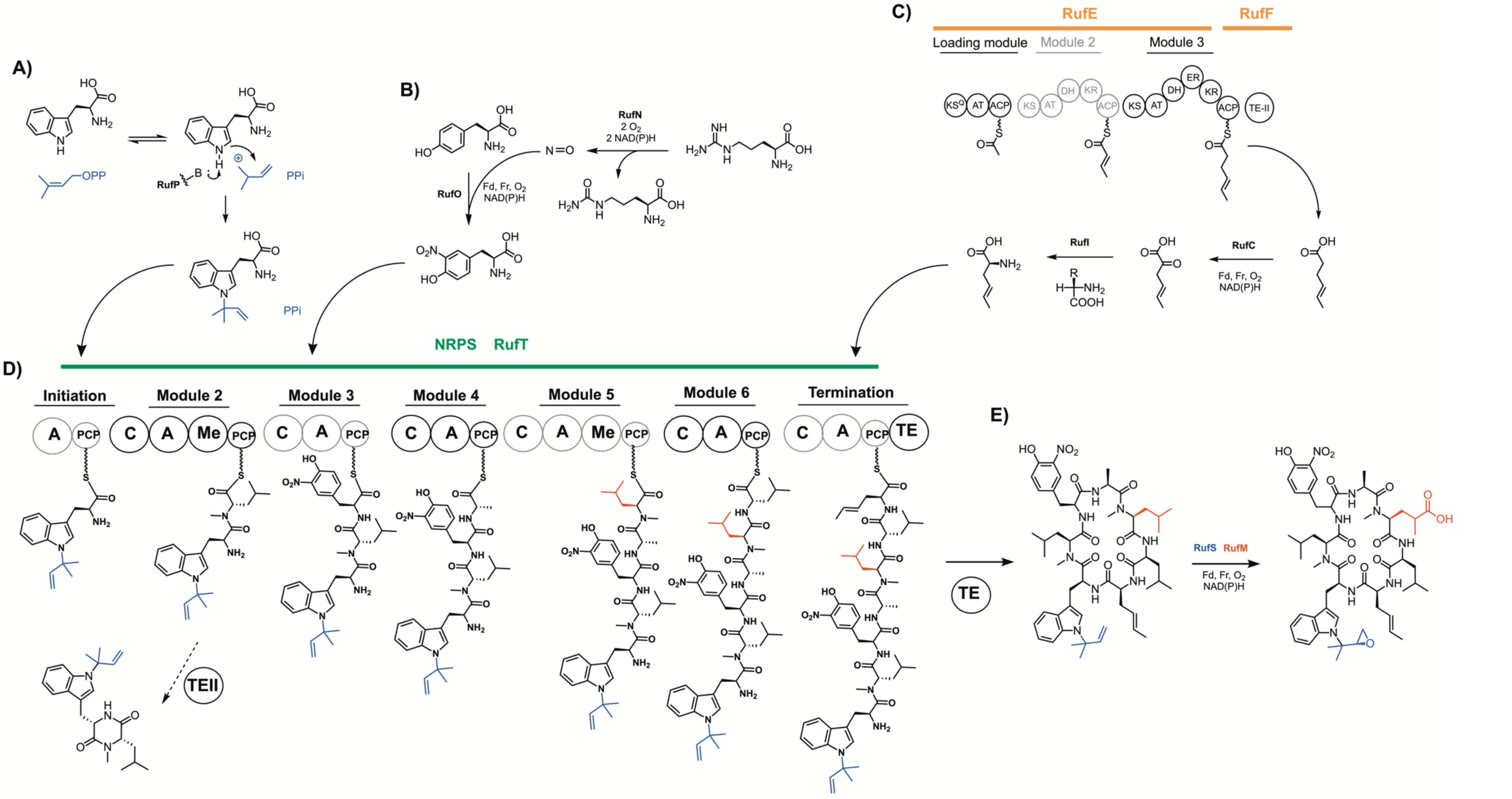

Supplementary Fig. S6. Biosynthesis of rufomycins and rufomyazine 11.

A) Biosynthesis of *N*-dimethylallyltryptophan by the RufP using DMAPP. B) Biosynthesis of 3-Nitro-L-tyrosine by the RufN/RufO nitro complex. C) Synthesis of L-2-amino-4-hexanoic acid, first as 4-hexenoic acid by the type-I PKS system RufE/RufF and modified by the monooxygenase RufC and then by the transaminase RufI. D) Synthesis of the cyclic peptide by the NRPS RufT. Rufomyazine is likely a shunt metabolite of the NRPS. E) the modifications of the scaffold by the tailoring enzymes RufS (blue) and RufM (red). [5, 6]

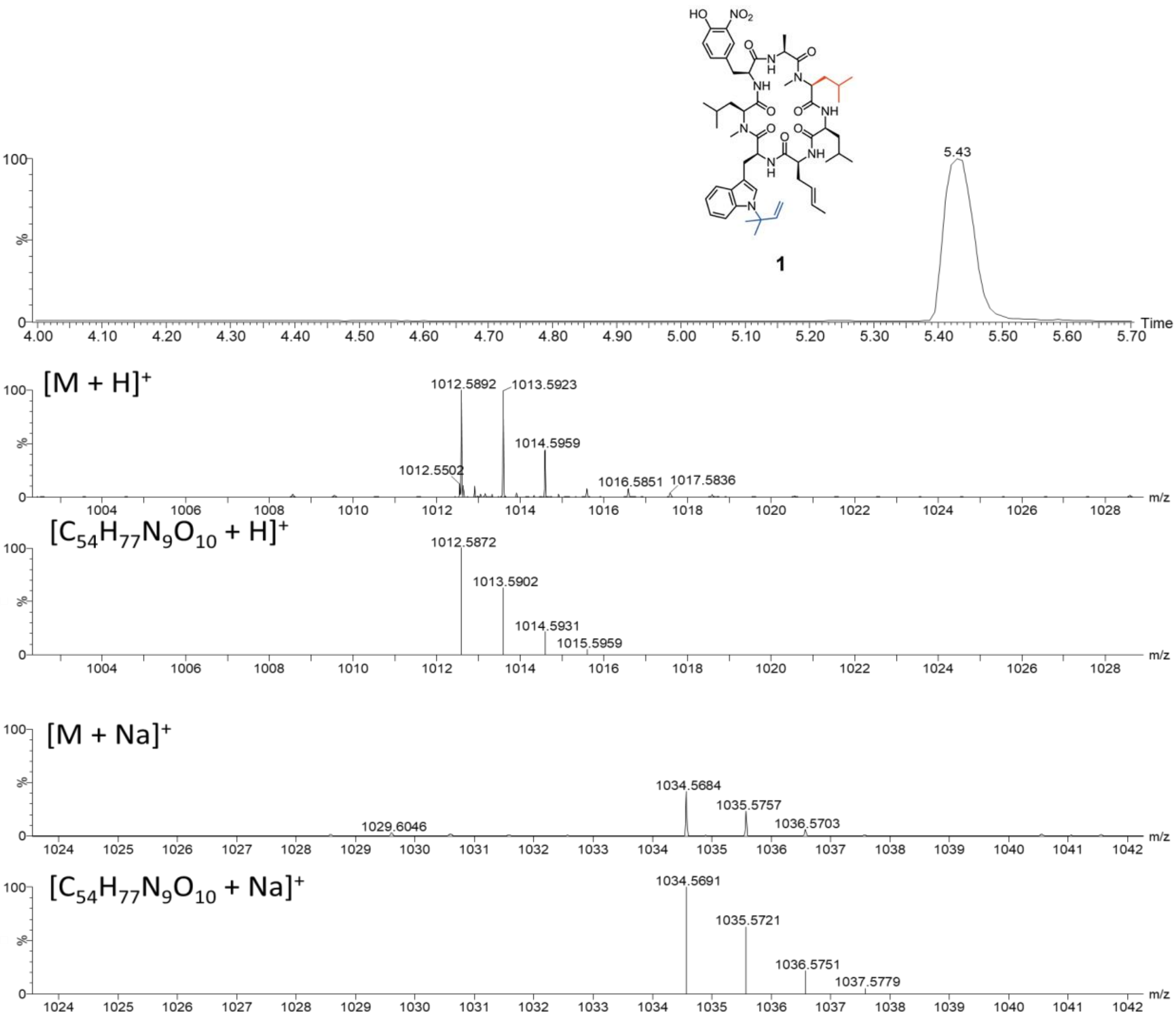

Supplementary Fig. S7. HRMS analysis of rufomycin B 1.

High resolution extracted Ion Chromatogram of rufomycin B **1**. Mass spectrum and simulated mass spectrum of rufomycin B **1**, <5 ppm. Mass spectrum and simulated mass spectrum of rufomycin B **1** sodium ion, <5 ppm.

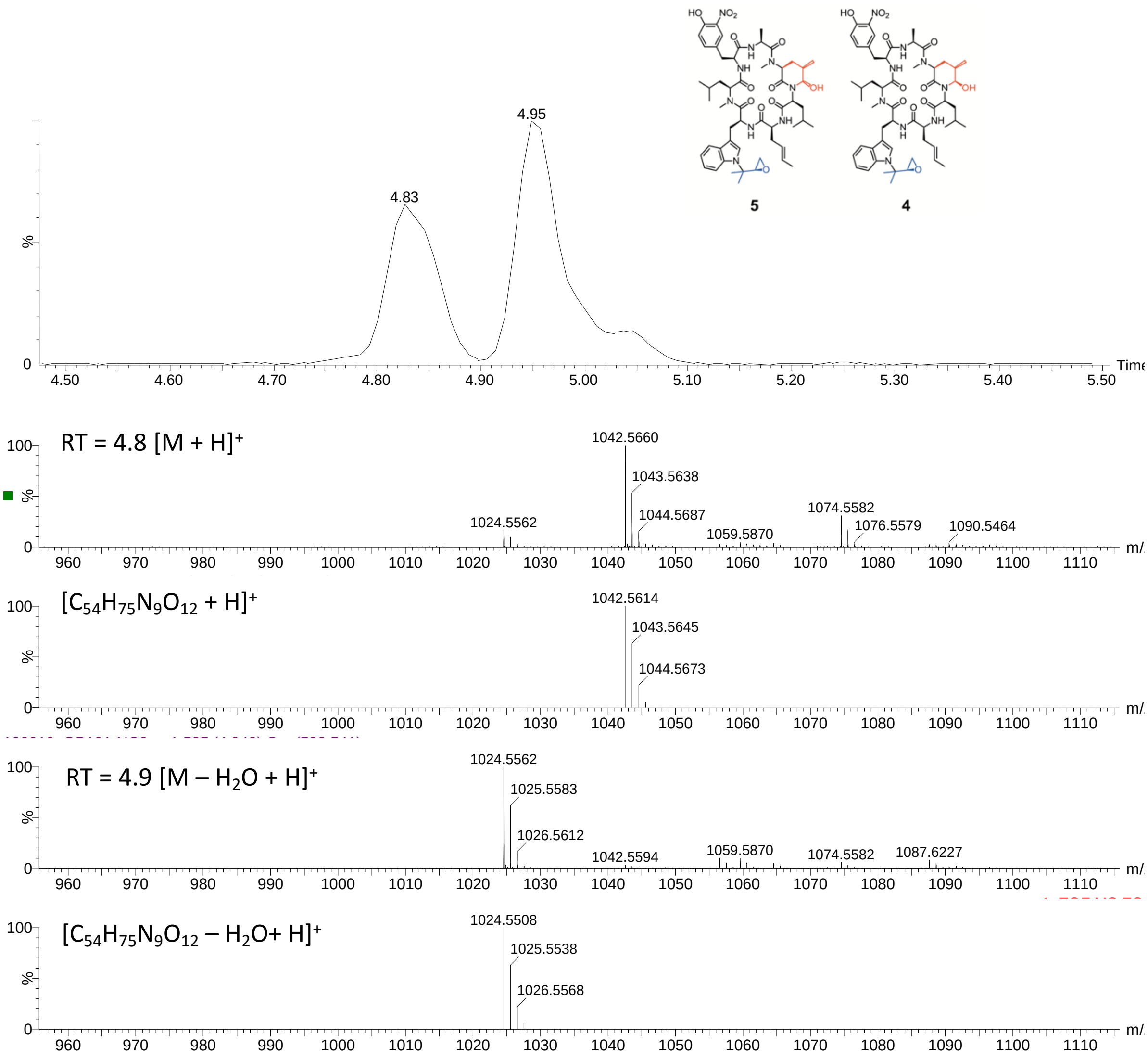

Supplementary Fig. S8. HRMS analysis of rufomycin A 4 & 5.

High resolution extracted Ion Chromatogram of rufomycin A **4, 5**. Mass spectrum and simulated mass spectrum of rufomycin A **4/5** (RT= 4.8 min) (RT= 4.9), >5 ppm. Mass spectrum and simulated mass spectrum of rufomycin A with hydrolysis ion, >5 ppm.

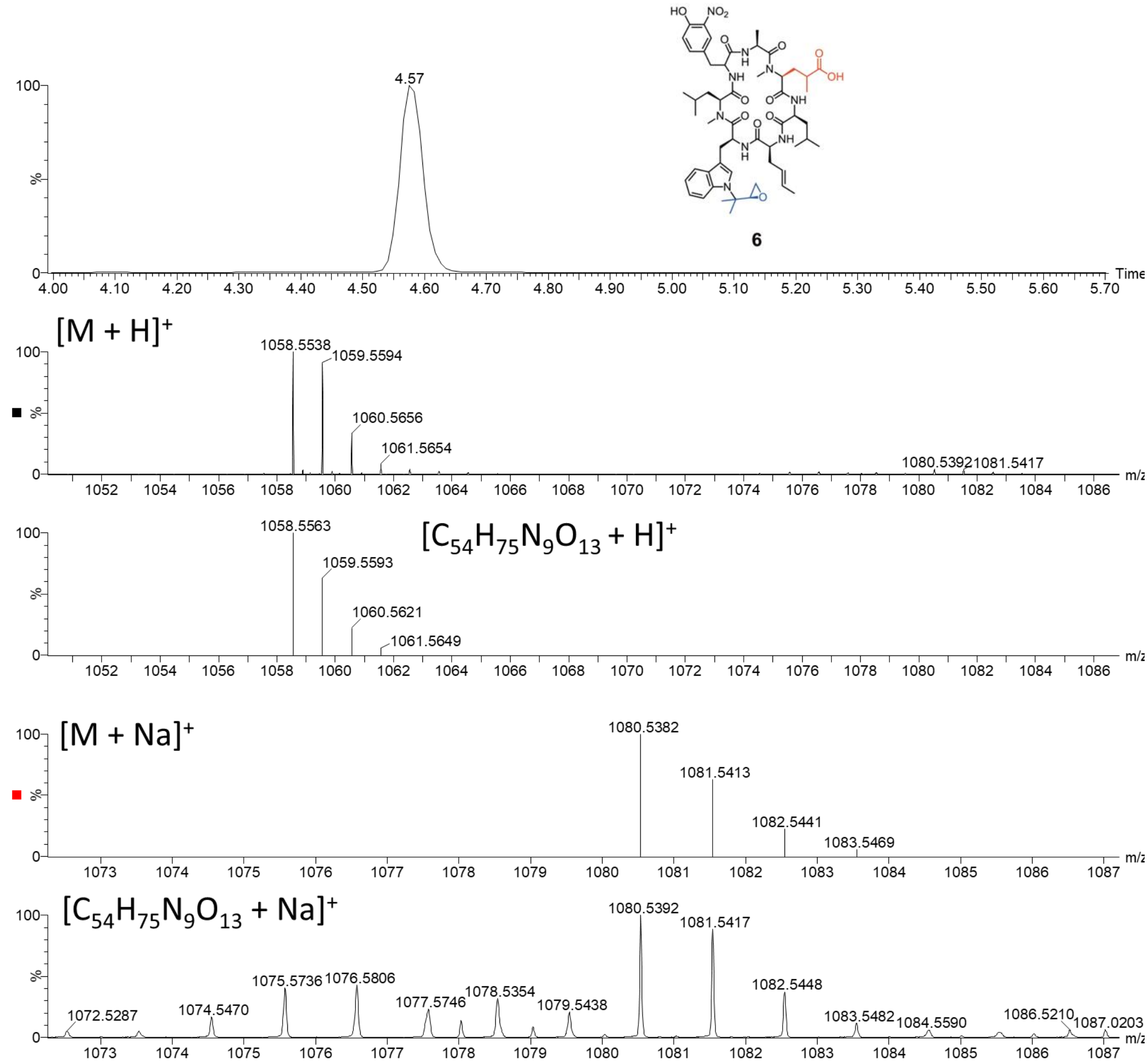

Supplementary Fig. S9. HRMS analysis of rufomycin C 6.

High resolution extracted Ion Chromatogram of rufomycin C **6**. Mass spectrum and simulated mass spectrum of rufomycin C **6**, >5 ppm. Mass spectrum and simulated mass spectrum of rufomycin C **6** sodium ion, >5 ppm.

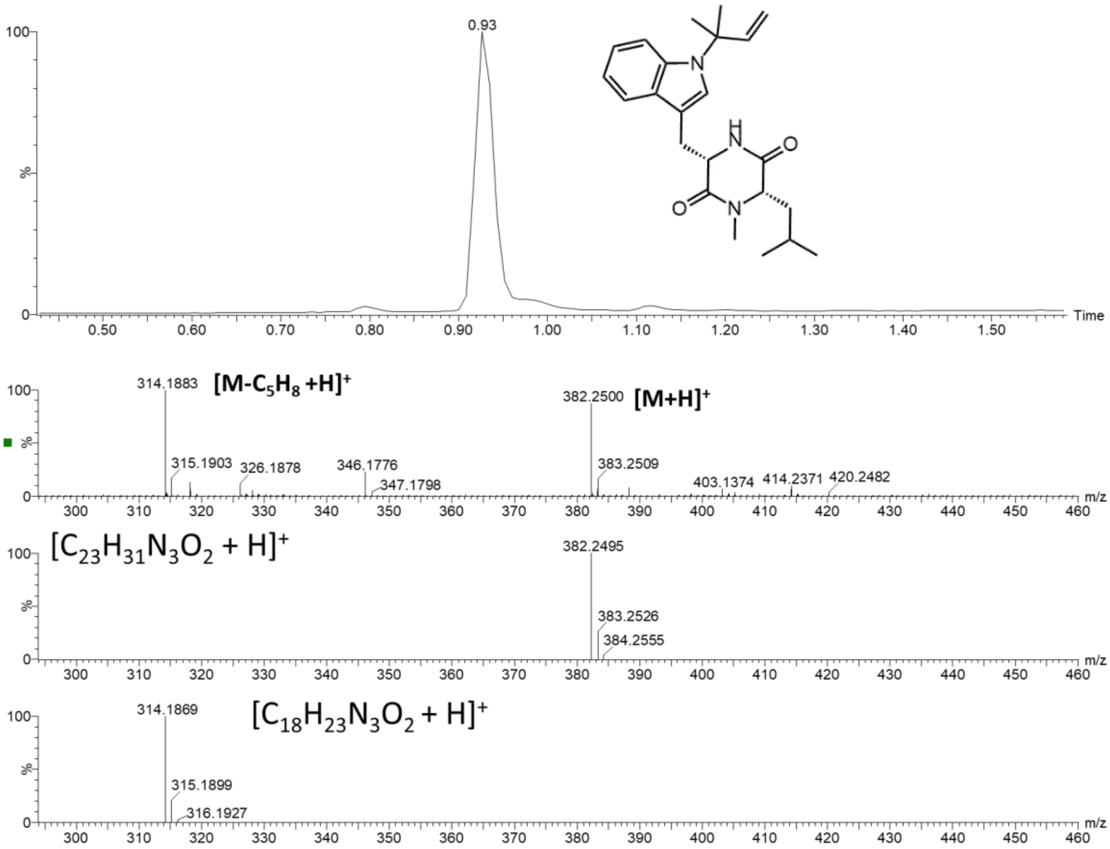

Supplementary Fig. S10. High resolution extracted Ion Chromatogram of rufomyazine 11 from *S. atratus* cultures in R5 media incubated for 6 days.

Extracted ion chromatogram of rufomyazine **11**. Mass spectrum and simulated mass spectra of rufomyazine and the ion after the loss of the 1-*N*-isoprenyl group of Trp.

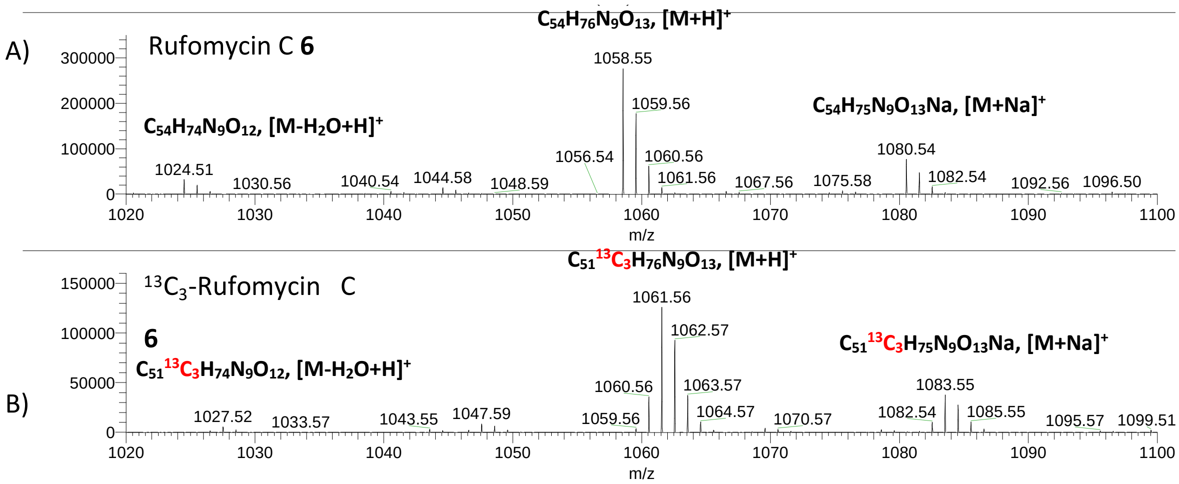

Supplementary Fig. S11. Rufomycin C 6 feeding experiment with ^{13}C-2-L-leucine.

A) Mass spectra of rufomycin C **6** from *S. atratus* culture extract supplemented with 10 mM L-leucine and B) supplemented with 10 mM ^{13}C-2-L-leucine.

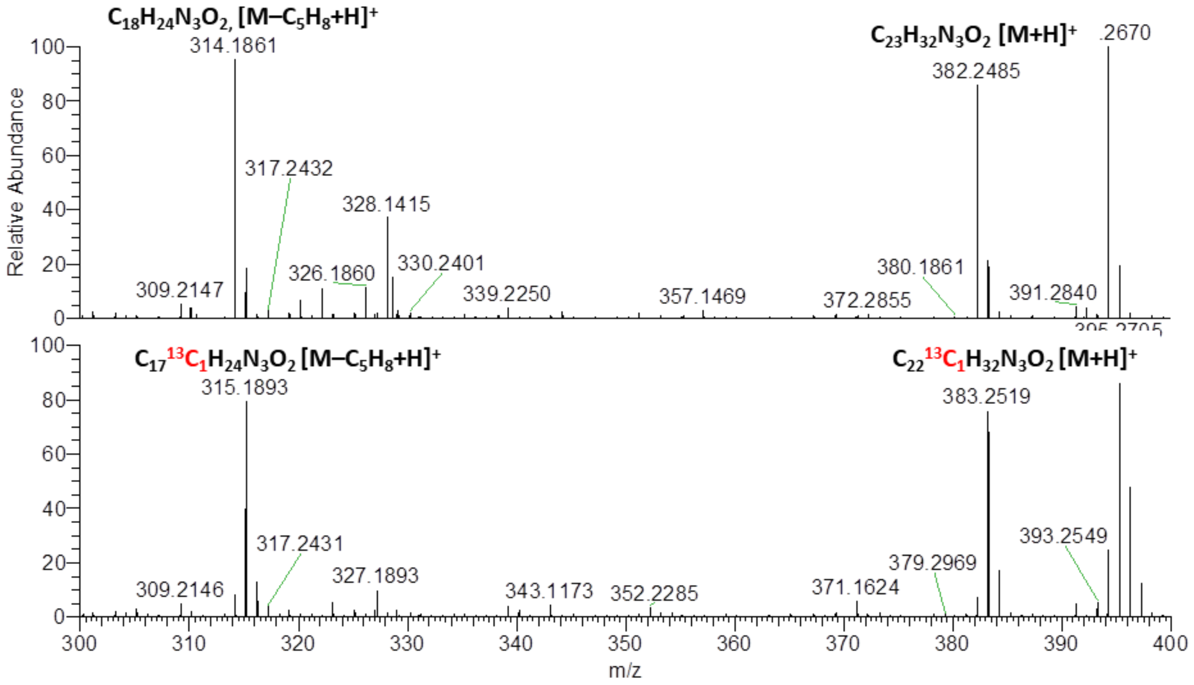

Supplementary Fig. S 12 Rufomyazine Feeding experiment with ^{13}C-2-L-leucine.

A) Mass spectra of rufomyazine from *S. atratus* culture extract supplemented with 10 mM L-leucine and B) supplemented with 10 mM ^{13}C-2-L-leucine.

Position	Ma *et al.* δH, multi. (*J* in Hz)	Rufomycin C (**6**) δH, multi. (*J* in Hz)
**Pre-Trp**		
2	4.93, dd (11.2, 4.3)	4.91, dd (11.2, 4.3)
3	3.45, dd (13.1, 4.3)	Overlapped
4	7.17, s	7.16, s
7	7.59, d (8.0)	7.56, d (7.9)
8	7.11, m	7.11, m
9	7,16, d (8.0)	7.14, d (8.3)
10	7.83, d (8.0)	7.80, d (8.5)
13	3.28, d (2.7)	3.28 overlapped
14	2.89, t, (4.3);	2.87, t (6.4)
	2.83, dd (4.4, 2.7)	2.82, dd (10.6, 5,2)
15	1.55, s	1.53, s
16	1.70, s	1.69, s
**$\Delta^{4,5}$ -NorLeu**		
18	4.64, m	4.54, m overlapped
19	2.78, m;	2.74 - 2.90, m overlapped
	2.51, m	2.46-2.53, m overlapped
20	5.25, m	5.21, m
21	5,55, m	5.50, m
22	1.59, d (6.7)	1.56, d (6.5)
**Leu**		
24	4.66, dd (11.5, 3.4)	4.60, dd (10.2, 5.1)
25	1.74, m	1.70 - 1.74, m overlapped
26	1.78, m	1.75 - 1.78, m overlapped
27	0.97, d (5.5)	0.95, d (5.7)
28	1.01, d (5.5)	0.98, d (5.7)

Position	Ma *et al.* δH, multi. (*J* in Hz)	Rufomycin C (**6**) δH, multi. (*J* in Hz)
**Modified-Nme-Leu**		
30	5.01, dd (9.0, 5.1)	4.98, dd (8.6, 5.3)
31	2.20, m;	2.18, m
	1.88, m	1.88, m
32	2.48, m	2.52, m
34	1.26, d (6.8)	1.24, d (6.9)
N-Me	2.75, s	2.73, s
**Ala**		
36	4.84, q (6.6)	Overlapped
37	1.30, d (6.6)	1.26, d (6.9)
**NO_2-Tyr**		
39	4.60, m	4.54, m overlapped
40	3.06, dd (13.0, 9.8);	3.02, dd (13.9, 10.8)
	2.73, m	2.72, m overlapped
42	7.80, d (2.1)	7.77, d (2.0)
45	7.10, d (8.5)	7.07, d (8.7)
46	7.43, dd (8.5, 2.1)	7.41, dd (8.6, 2.1)
**NMeLeu**		
48	4.68, dd (11.6, 2.9)	4.65, dd (11,6, 3.1)
49	1.60, m	1.63, m overlapped
50	1.11, m	1.15, m overlapped
51	0.20, d (6.6)	0.15, d (6.6)
52	0.40, d (6.6)	0.37, d (6.6)
N-Me	2.69, s	2.67, s

Supplementary Table S1. ^{1}H NMR spectroscopic data of rufomycin C (6) and the reported data for the same compound.

The reported data published by Ma *et al.*[6] was used as reference to confirm the identity of the final, fully oxidised compound of rufomycin biosynthesis in *S. atratus.*

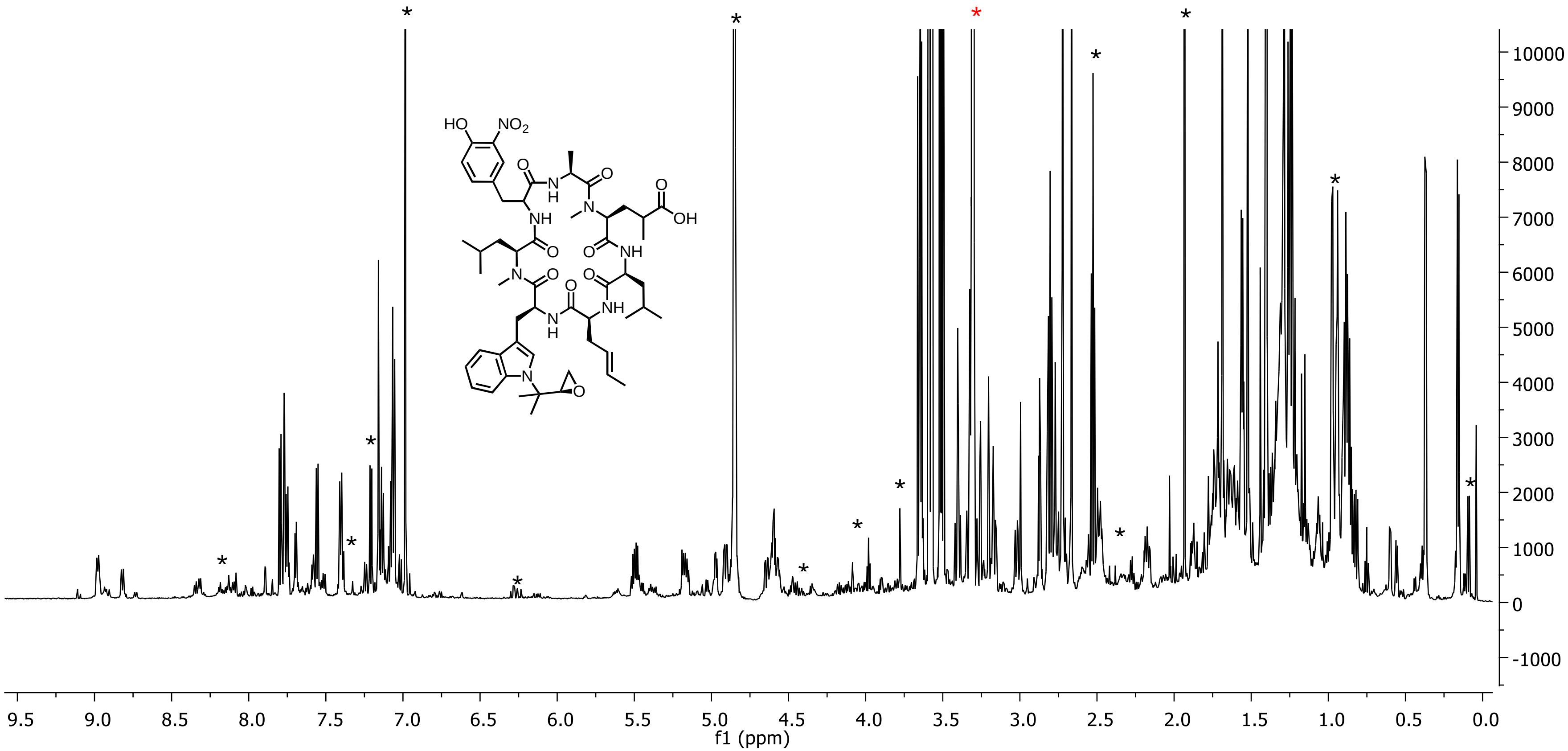

Supplementary Fig. S13. ^{1}H NMR spectrum of rufomycin C 6 in CD_3OD at 700 MHz.

Isolated mass ~0.5mg. Impurities are indicated with the * symbol and solvent in red. The spectrum matches the published data by Ma et al, 2017 [6].

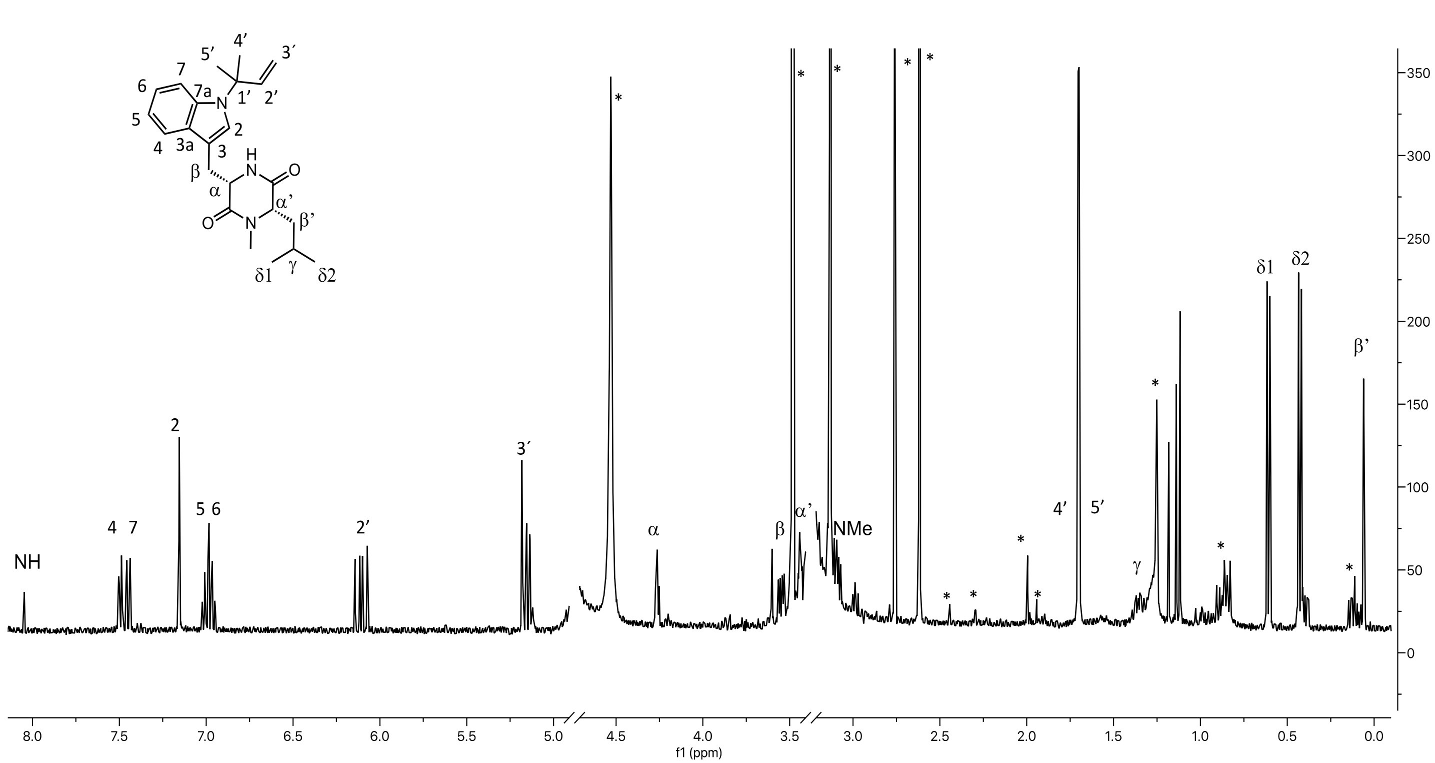

Position	Rufyazine **11** d_H, multi. (*J* in Hz)
	Pre-Trp
a	4.26, dt
b	3.37, overlapped
	3.11, overlapped
2	7.15, s
4	7.49, m overlapped
5	6.99, m overlapped
6	6.98, m overlapped
7	7.45, m overlapped
2'	6.11, dd (17.4, 10.9)
3'	5.17, dd (10.1, 0.7)
	5.13, dd (12, 0.8)
4'	1.70,s
5'	1.69, s
NH	8.05, s
	Nme-Leu
a	3.55, dd (9.3, 4.0)
b	0.41, overlapped
	0.11
g	1.37, m
d1	0.61, d (6.5)
d2	0.43, d (6.7)
NMe	2.76, s

Supplementary Fig. S14. ^{1}H NMR spectrum of rufomyazine 11 in CD_3OD at 400 MHz.

Left: Impurities are indicated with the * symbol. The spectrum matches the published data by Choules et al [7]. Right: ^{1}H NMR spectroscopic data of rufomyazine **11**.

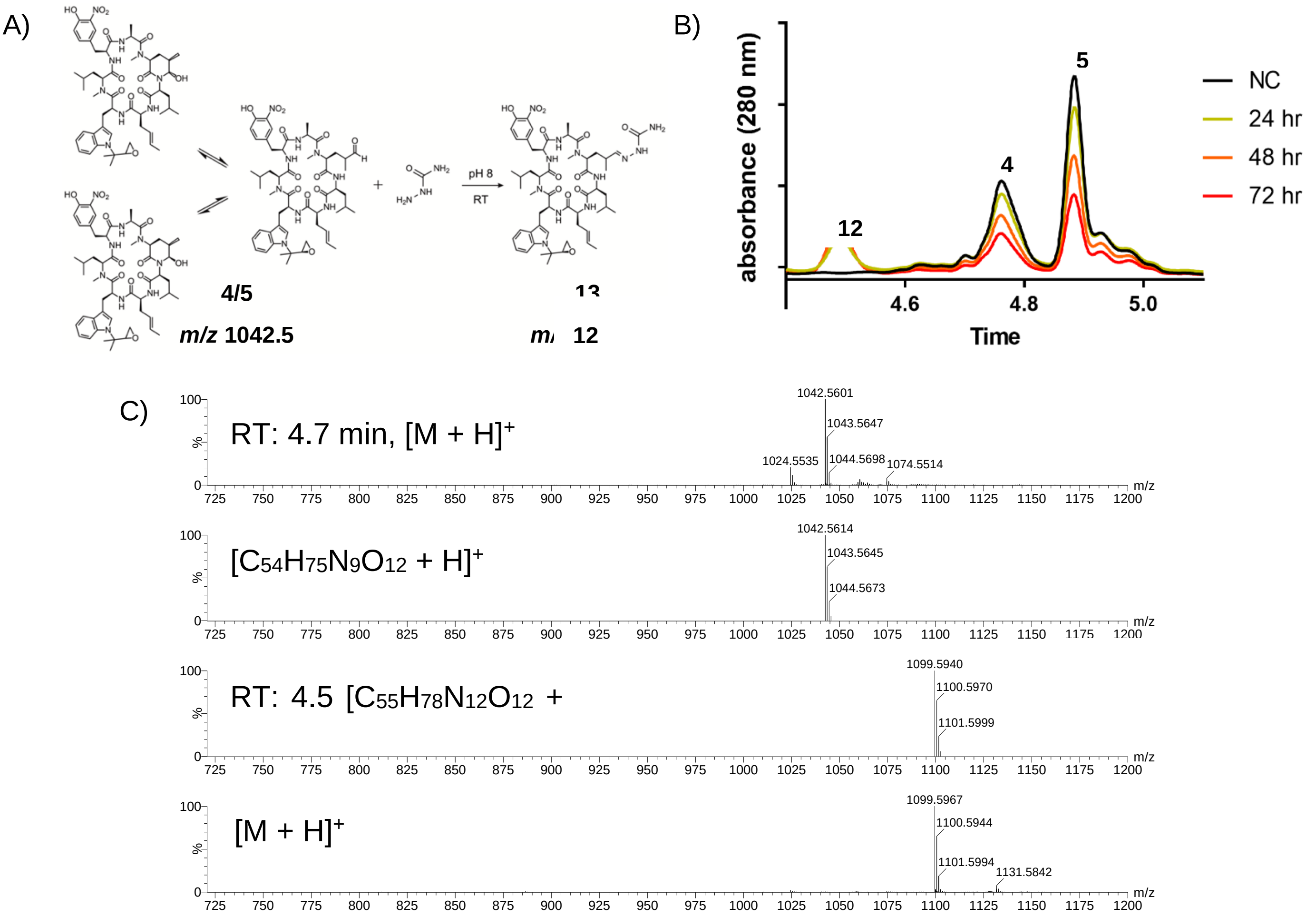

Supplementary Fig. S15. Reaction of Rufomycin B 4/5 with Semicarbazide.

A) Scheme of reaction using method from Li *et al* [2]. B) Time course of reaction of aldehyde rufomycin and semicarbazide in Tris pH 8 at room temperature for up to 72 hrs. C) Mass spectrum and simulated mass spectrum of rufomycin A **4,5**. D) Mass spectrum and simulated mass spectrum of the adduct of rufomycin-semicarbazide **12.**

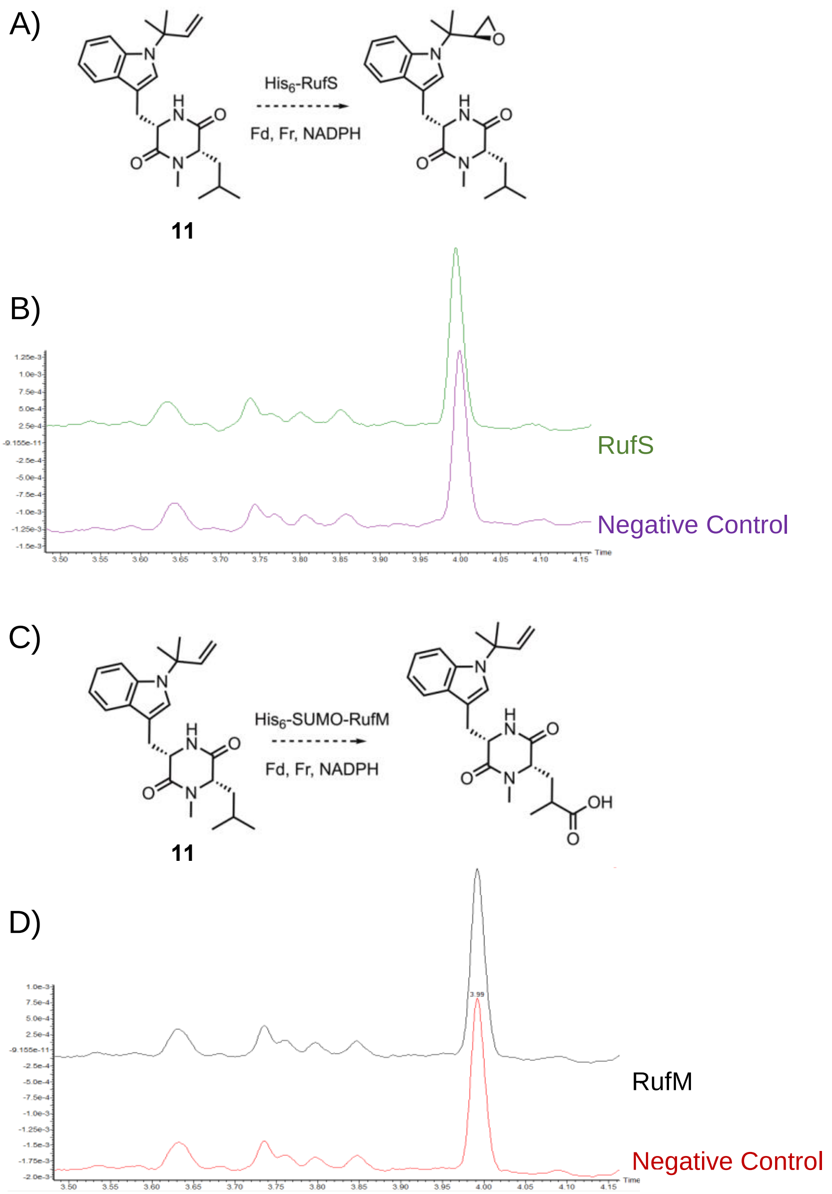

Supplementary Fig. S16. Reaction of RufM and RufS with rufomyazine.

A) Epoxidation reaction carried out by His$_6$-RufS of rufomyazine **11**. B) UV-vis traces at 280nm of epoxidation reaction after 3 hr incubation and negative control incubated with inactivated enzyme. C) Oxidation reaction carried out by His$_6$-SUMO-RufM of rufomyazine **11.** D) UV-vis traces at 280nm of oxidation reaction after 3 hr incubation and negative control incubated with inactivated enzyme.

Supplementary Tables

Time (min)	% Water +0.1% TFA	% Acetonitrile +0.1% TFA
0	95	5
25	0	100
30	0	100
35	95	5
40	95	5

Supplementary Table S2. The Prep-HPLC mobile phase gradient profile used for purification of the crude rufomycin extract, 20mL min^{-1} flow rate.

Time (min)	% Water +0.1% TFA	% Acetonitrile +0.1% TFA
0	100	0
1	90	10
30	30	70
31	10	90
35	10	90
36	100	0
42	100	0

Supplementary Table S3. The analytical HPLC mobile phase gradient profile used for the purification of the fraction of rufomycin extract, 1mL min^{-1} flow rate and LCMS. Formic acid was used instead of TFA in LCMS runs.

Time (min)	% Water +0.1% FA	% Acetonitrile +0.1% FA
0	95	5
0.5	95	5
4.5	5	95
5.5	5	95
6.0	95	5
6.5	95	5

Supplementary Table S4. HPLC mobile phase gradient profile used for HRMS, 0.400 mL min^{-1} flow rate.

Primer sequence 5' – 3'	F/R	Annealing Temp (°C)	Purpose
CACCGTGCTCAACAGCGAGAATG	Fwd	57.0	For *rufM* amplification
TCAGAGGGTGACCGGGAG	Rev	57.0	For *rufM* amplification
ACCACCAATCTGTTCTCTGTG	Rev	65.4	For Assembly of GPO151-M002 containing His_6-SUMO-RufM
ACAGAGAACAGATTGGTGGTGTGC TCAACAGCGAGAATGC	Fwd	67.9	For Assembly of GPO151-M002 containing His_6-SUMO-RufM
CTTTGTTAGCAGCCGGATCCTCAGA GGGTGACCGGGAG	Rev	67.9	For Assembly of GPO151-M002 containing His_6-SUMO-RufM
GGATCCGGCTGCTAACAAAG	Fwd	65.4	For Assembly of GPO151-M002 containing His_6-SUMO-RufM

Supplementary Table S5. Primers used in this study

Cycles	Temperature (°C)	Time
**1**	95	2 min
**30**	95	30 sec
	57	45 sec
	72	2 min
**1**	72	15 min

Supplementary Table S6. PCR cycle parameters for the amplification of the PCR fragments of *rufM* from genomic DNA.

Cycles	Temperature (°C)	Time
**1**	68.5	1 min
**30**	98	10 sec
	**X**	20 sec
	72	3 min
**1**	72	7 min

Supplementary Table S7. PCR cycle parameters for the amplification of the PCR fragments used during the cloning r*ufM* to produce plasmid GPO151-M002.

X = annealing temp according to Supplementary Table 4.

**Predicted monoisotopic m/z for [M + H]$^+$**	**Rufomycin B 1** 1012.5872 m/z		**Rufomycin 2** 1028.5820 m/z	
**Chemical Formula**	$C_{54}H_{77}N_9O_{10}$		$C_{54}H_{77}N_9O_{11}$	
**Sample**	Observed m/z	Error (ppm)	Observed m/z	Error (ppm)
Reaction 1hr incubation	1012.5892	1.975	1028.587	4.86
Reaction 2hr incubation	1012.5892	1.975	1028.587	4.86
Reaction 3hr incubation	1012.5892	1.975	1028.587	4.86
Negative Control	1012.5892	1.975		

Supplementary Table S8. Epoxidation of rufomycin B 1 by His6-RufS.

Mass measurements taken from the monoisotopic peaks after spectra were background subtracted, smoothed and centred, errors (ppm).

**Predicted monoisotopic m/z for [M + H]$^+$**	**Rufomycin B 1** 1012.5872 m/z		**Rufomycin 7, 8, 9** 1026.5664 m/z		**Rufomycin 10** 1042.5614 m/z	
**Chemical Formula**	$C_{54}H_{77}N_9O_{10}$		$C_{54}H_{75}N_9O_{11}$		$C_{54}H_{75}N_9O_{12}$	
**Sample**	Observed m/z	Error (ppm)	Observed m/z	Error (ppm)	Observed m/z	Error (ppm)
Reaction 2hr incubation	1012.5827	-4.444	1026.5599	-6.33	1042.5616	0.19
Reaction 3hr incubation	1012.5827	-4.444	1026.5599	-6.33	1042.5616	0.19
Negative Control	1012.5827	-4.444				

Supplementary Table S9. Oxidation of rufomycin B 1 by His$_6$-SUMO-RufM.

Mass measurements taken from the monoisotopic peaks after spectra were background subtracted, smoothed and centred, errors (ppm).

**Predicted monoisotopic m/z for [M + H]$^+$**	**Rufomycin B (1)** 1012.5872 m/z		**Rufomycin (13)** 1028.5820 m/z		**Rufomycin (8/9)** 1026.5664 m/z		**Rufomycin (10)** 1042.5614 m/z	
**Chemical Formula**	$C_{54}H_{77}N_9O_{10}$		$C_{54}H_{77}N_9O_{11}$		$C_{54}H_{79}N_9O_{11}$		$C_{54}H_{75}N_9O_{12}$	
**Sample**	Observed m/z	Error (ppm)	Observed m/z	Error (ppm)	Observed m/z	Error (ppm)	Observed m/z	Error (ppm)
45 μM Fd / 4 U Fr	1012.5892	1.975	1028.5817	12.3	1026.5599	-6.33	1042.5614	-2.362
22.5 μM Fd / 2 U Fr	1012.5892	1.975	1028.5793	-2.62	1026.5654	0.974	1042.5614	-2.362
11.3 μM Fd / 1 U Fr	1012.5892	1.975	1028.5793	-2.62	1026.5654	0.974	1042.5614	-2.362
5.7 μM Fd / 0.5 U Fr	1012.5892	1.975	1028.5793	-2.62	1026.5654	0.974	1042.5614	-2.362
Negative Control	1012.5892	1.975						

Supplementary Table S10. Oxidation of rufomycin B1 by His$_6$-SUMO-RufM.

Mass measurements taken from the monoisotopic peaks after spectra were background subtracted, smoothed and centred, errors (ppm).

Predicted monoisotopic m/z for [M + H]$^+$	Rufomycin A (4,5) 1042.5614 m/z		Rufomycin C (6) 1058.5563 m/z	
**Chemical Formula**	$C_{54}H_{75}N_9O_{12}$		$C_{54}H_{75}N_9O_{13}$	
**Sample**	Observed m/z	Error (ppm)	Observed m/z	Error (ppm)
Rufomycin A **4, 5**	1042.5616	0.192		
Negative Control	1042.5616	0.192		
RufM reaction			1058.5538	-2.362
Rufomycin C **6**			1058.5538	-2.362

Supplementary Table S11. Oxidation of rufomycin A 4/5 by His$_6$-SUMO-RufM.

Mass measurements taken from the monoisotopic peaks after spectra were background subtracted, smoothed and centred, errors (ppm).

Predicted monoisotopic m/z for [M + H]$^+$	Rufomycin B (1) 1012.5872 m/z		Rufomycin (2) 1028.5820 m/z		Rufomycin (10) 1042.5614 m/z		Rufomycin C (6) 1058.5563 m/z	
**Chemical Formula**	$C_{54}H_{77}N_9O_{10}$		$C_{54}H_{77}N_9O_{11}$		$C_{54}H_{75}N_9O_{12}$		$C_{54}H_{75}N_9O_{13}$	
**Sample**	Observed m/z	Error (ppm)	Observed m/z	Error (ppm)	Observed m/z	Error (ppm)	Observed m/z	Error (ppm)
Rufomycin B **1**	1012.5892	1.975						
Reaction with RufM	1012.5892	1.975			1042.5616	0.192		
Reaction with RufS			1028.5804	-1.555			1058.5538	-2.362
Rufomycin C **6**							1058.5538	-2.362

Supplementary Table S12. Sequential reaction 1: oxidation reaction of rufomycin B 1 by RufM followed by epoxidation reaction by RufS.

Mass measurements taken from the monoisotopic peaks after spectra were background subtracted, smoothed and centred, errors (ppm).

**Predicted monoisotopic m/z for [M + H]+**	**Rufomycin B (1)** 1012.5872 m/z		**Rufomycin (2)** 1028.5820 m/z		**Rufomycin (10)** 1042.5614 m/z		**Rufomycin C (6)** 1058.5563 m/z	
**Chemical Formula**	$C_{54}H_{77}N_9O_{10}$		$C_{54}H_{77}N_9O_{11}$		$C_{54}H_{75}N_9O_{12}$		$C_{54}H_{75}N_9O_{13}$	
**Sample**	Observed m/z	Error (ppm)	Observed m/z	Error (ppm)	Observed m/z	Error (ppm)	Observed m/z	Error (ppm)
Rufomycin B **1**	1012.5892	1.975						
Reaction with RufS	1012.5892	1.975	1028.5804	-1.555				
Reaction with RufM					1042.5616	0.192	1058.5538	-2.362
Rufomycin C **6**							1058.5538	-2.362

Supplementary Table S13. Sequential reaction 2: epoxidation reaction of rufomycin B 1 by RufS followed by oxidation reaction by RufM.

Mass measurements taken from the monoisotopic peaks after spectra were background subtracted, smoothed and centred, errors (ppm).

**Predicted monoisotopic m/z for [M + H]+**	**Rufomycin B (1)** 1012.5872 m/z		**Rufomycin C (6)** 1058.5563 m/z	
**Chemical Formula**	$C_{54}H_{77}N_9O_{10}$		$C_{54}H_{75}N_9O_{13}$	
**Sample**	Observed m/z	Error (ppm)	Observed m/z	Error (ppm)
Rufomycin B **1**	1012.5827	-4.444		
Negative Control	1012.5827	-4.444		
RufS:RufM 1:1	1012.5827	-4.444	1058.5538	-2.362
RufS:RufM 1:1 (doubled Fr, Fd)	1012.5827	-4.444	1058.5538	-2.362
RufS:RufM 1:2	1012.5827	-4.444	1058.5538	-2.362
RufS:RufM 2:1	1012.5827	-4.444	1058.5538	-2.362
Rufomycin C **6**			1058.5538	-2.362

Supplementary Table S14. RufM and RufS simultaneous reactions with rufomycin B 1.

Mass measurements taken from the monoisotopic peaks after spectra were background subtracted, smoothed and centred, errors (ppm).

Predicted monoisotopic m/z for [M + H]+	Rufomycin B (1) 1012.5872 m/z		Rufomycin (2) 1028.5820 m/z		Rufomycin (4/5) 1042.5614 m/z		Rufomycin C (6) 1058.5563 m/z	
**Chemical Formula**	$C_{54}H_{77}N_9O_{10}$		$C_{54}H_{77}N_9O_{11}$		$C_{54}H_{75}N_9O_{12}$		$C_{54}H_{75}N_9O_{13}$	
**Sample**	Observed m/z	Error (ppm)	Observed m/z	Error (ppm)	Observed m/z	Error (ppm)	Observed m/z	Error (ppm)
90 μM Fd /0.16 U Fr							1058.5538	-2.362
67.5 μM Fd /0.12 U Fr					1042.5616	0.192	1058.5538	-2.362
45 μM Fd / 0.08 U Fr	1012.5892	1.975	1028.5817	1.02	1042.5616	0.192	1058.5538	-2.362
22.5 μM Fd /0.04 U Fr	1012.5892	1.975	1028.5817	1.02				
11.3 μM Fd /0.02 U Fr	1012.5892	1.975						
Negative control	1012.5892	1.975						

Supplementary Table S15. RufM and RufS simultaneous reactions with rufomycin B 1 and different concentrations of electron donors Fd and Fr.

Mass measurements taken from the monoisotopic peaks after spectra were background subtracted, smoothed and centred, errors (ppm).

Supporting references

1. T. B. Kieser, M.J. Bibb, M. J. Buttner, K. F.Chater and D. A. Hopwood, Practical Streptomyces Genetics *John Innes Foundation, Norwich*, 2000.
2. F. Li, J. Lu and X. Ma, *Chem. Res. Toxicol.*, 2011, **24**, 744-751.
3. G. Bertani, *J. Bacteriol.*, 1951, **62**, 293-300.
4. U. T. Nguyen, L. Bittova, M. M. Muller, B. Fierz, Y. David, B. Houck-Loomis, V. Feng, G. P. Dann and T. W. Muir, *Nat Methods*, 2014, **11**, 834-840.
5. H. Tomita, Y. Katsuyama, H. Minami and Y. Ohnishi, *J. Biol. Chem.*, 2017, 2017, **292**,15859.
6. J. Ma, H. Huang, Y. Xie, Z. Liu, J. Zhao, C. Zhang, Y. Jia, Y. Zhang, H. Zhang, T. Zhang and J. Ju, *Nat. Commun*, 2017, **8**, 391.
7. M. P. Choules, L. L. Klein, D. C. Lankin, J. B. McAlpine, S. H. Cho, J. Cheng, H. Lee, J. W. Suh, B. U. Jaki, S. G. Franzblau and G. F. Pauli, *J. Org. Chem.*, 2018, **83**, 6664-6672.
